# Molecular Insights into an Antibiotic Enhancer Action of New Morpholine-Containing 5-Arylideneimidazolones in the Fight against MDR Bacteria

**DOI:** 10.3390/ijms22042062

**Published:** 2021-02-19

**Authors:** Aneta Kaczor, Karolina Witek, Sabina Podlewska, Veronique Sinou, Joanna Czekajewska, Ewa Żesławska, Agata Doroz-Płonka, Annamaria Lubelska, Gniewomir Latacz, Wojciech Nitek, Markus Bischoff, Sandrine Alibert, Jean-Marie Pagès, Claus Jacob, Elżbieta Karczewska, Jean-Michel Bolla, Jadwiga Handzlik

**Affiliations:** 1 Department of Technology and Biotechnology of Drugs, Jagiellonian University, Medical College, ul. Medyczna 9, 30-688 Krakow, Poland; aneta.kaczor@doctoral.uj.edu.pl (A.K.); karolina.witek@uj.edu.pl (K.W.); sabina.podlewska@uj.edu.pl (S.P.); a.doroz-plonka@uj.edu.pl (A.D.-P.); annamarialubelska@outlook.com (A.L.); glatacz@cm-uj.krakow.pl (G.L.); 2 Department of Pharmaceutical Microbiology, Jagiellonian University, Medical College, ul. Medyczna 9, 30-688 Krakow, Poland; j.czekajewska@uj.edu.pl (J.C.); elzbieta.karczewska@uj.edu.pl (E.K.); 3 UMR_MD1, U-1261, Aix Marseille Univ, INSERM, SSA, MCT, Faculté de Pharmacie, 27 Bd Jean Moulin, 13005 Marseille, France; veronique.sinou@univ-amu.fr (V.S.); sandrine.alibert@univ-amu.fr (S.A.); jean-marie.pages@univ-amu.fr (J.-M.P.); jean-michel.bolla@univ-amu.fr (J.-M.B.); 4 Institute for Medical Microbiology and Hygiene, Saarland University, D-66421 Homburg/Saar, Germany; Markus.Bischoff@uniklinikum-saarland.de; 5 Division of Bioorganic Chemistry, School of Pharmacy, Saarland University, Campus B 2.1, D-66123 Saarbruecken, Germany; c.jacob@mx.uni-saarland.de; 6 Maj Institute of Pharmacology, Polish Academy of Sciences, Department of Medicinal Chemistry, ul. Smętna 12, 31-343 Krakow, Poland; 7 Pedagogical University of Cracow, Institute of Biology, ul. Podchorążych 2, 30-084 Krakow, Poland; ewa.zeslawska@up.krakow.pl; 8 Faculty of Chemistry, Jagiellonian University, ul. Gronostajowa 2, 30-387 Krakow, Poland; wojciech.nitek@uj.edu.pl

**Keywords:** 5-arylideneimidazolones, multidrug resistance, antibiotic adjuvant, *Staphylococcusaureus*, MRSA, *Klebsiella aerogenes*, PBP2a, AcrAB-TolC, molecular docking, RTE assay

## Abstract

In the search for an effective strategy to overcome antimicrobial resistance, a series of new morpholine-containing 5-arylideneimidazolones differing within either the amine moiety or at position five of imidazolones was explored as potential antibiotic adjuvants against Gram-positive and Gram-negative bacteria. Compounds (**7**–**23**) were tested for oxacillin adjuvant properties in the Methicillin-susceptible *S. aureus* (MSSA) strain ATCC 25923 and Methicillin-resistant *S. aureus* MRSA 19449. Compounds **14**–**16** were tested additionally in combination with various antibiotics. Molecular modelling was performed to assess potential mechanism of action. Microdilution and real-time efflux (RTE) assays were carried out in strains of *K. aerogenes* to determine the potential of compounds **7**–**23** to block the multidrug efflux pump AcrAB-TolC. Drug-like properties were determined experimentally. Two compounds (**10**, **15**) containing non-condensed aromatic rings, significantly reduced oxacillin MICs in MRSA 19449, while **15** additionally enhanced the effectiveness of ampicillin. Results of molecular modelling confirmed the interaction with the allosteric site of PBP2a as a probable MDR-reversing mechanism. In RTE, the compounds inhibited AcrAB-TolC even to 90% (**19**). The 4-phenylbenzylidene derivative (**15**) demonstrated significant MDR-reversal “dual action” for *β*-lactam antibiotics in MRSA and inhibited AcrAB-TolC in *K. aerogenes*. **15** displayed also satisfied solubility and safety towards CYP3A4 in vitro.

## 1. Introduction

### 1.1. Bacterial Multidrug Resistance

The discovery of the first antibiotic by Alexander Fleming in 1928 was a great breakthrough in the treatment of bacterial infectious diseases. Rather unexpectedly, bacterial infections revived to become a global threat once again due to the emergence, spread and persistence of multidrug resistant (MDR) bacteria all over the world [1,2,3,4]. MDR bacteria have caused already a few epidemics and this problem is increasing rapidly [5]. Although this issue has raised a lot of attention in recent years, all policies and incentives that have been proposed to combat MDR brought only a little progress yet, and the current lack of solutions is likely to exert major consequences on our healthcare system and may also affect social and economic fields [6]. Thus, it is crucial to search for new antimicrobial drugs or other strategies able to overcome MDR [2,4,7,8]. Such strategies include, for instance, the use of antibodies, bacteriophages, and antibiotic adjuvant therapy [9,10]. In this context, the development of adjuvant molecules able to restore antibacterial activity of marketed antibiotics is an attractive strategy [10,11,12]. The great efficacy of chemosensitizers is related to inhibition of various mechanisms of bacterial resistance e.g., permeation across outer membrane, efflux pumps, modification of drug target, and enzymatic inactivation of drugs [8,10].

### 1.2. AcrAB-TolC Efflux Pump

Among bacterial defense strategies, active efflux is a widespread and simple mechanism contributing to the first line of bacterial defense [6]. Membrane transport proteins, e.g., efflux pumps, can extrude intracellularly acting antibiotics but also many other structurally diverse substances, e.g., toxins, bile salts, dyes, detergents, and organic solvents. Moreover, they are one of the major contributors to clinical MDR in Gram-positive and Gram-negative bacteria [13,14,15,16]. The tripartite system AcrAB-TolC, a member of the Resistance-Nodulation-Cell Division (RND) family, is one of the most important efflux pumps utilized by Gram-negative MDR bacteria, which confers resistance to a broad range of antibiotics, such as *β*-lactams, fluoroquinolones, macrolides, and tetracyclines [13]. This pump is composed of the following proteins: a transport protein located in the inner membrane (AcrB), a membrane fusion protein (AcrA), and an outer membrane protein (TolC) [14], which form a complex able to export intracellularly localized cargo into the extracellular milieu via an electrochemical gradient [13]. Antibiotic adjuvants able to block efflux-mediated mechanisms of resistance are called efflux pump inhibitors (EPIs) [10]. Richter et al. pointed out that potential EPIs, which probably accumulate in Gram-negative bacteria, should (i) possess an amine (preferably primary amine), (ii) be amphiphilic, (iii) be rigid, and (iv) possess low globularity [17].

### 1.3. Penicillin Binding Protein (PBP)

In the group of Gram-positive bacteria, multidrug-resistant variants of *Staphylococcus aureus* are of major concern. The bacterium is responsible for a wide range of infections [18]. This ability is connected to various virulence factors, e.g., enzymes and toxins [18]. Nowadays, *S. aureus* is one of the “superbugs” due to the acquisition of multiple resistance determinants, and a lot of attention is drawn to methicillin-resistant *S. aureus* (MRSA) [4,18,19]. MRSA harbors a mobile genetic element called staphylococcal cassette chromosome mec (SCCmec) within their chromosome, which includes among others the genes mecA or mecC, encoding for an alternative penicillin binding protein, PBP2a, which has reduced affinity for *β*-lactam antibiotics [8,20].

PBP and its modification PBP2a are enzymes which bind to bacterial membrane. They catalyze transpeptidation and transglycosylation reactions, which are necessary during bacterial cell wall biosynthesis [21,22]. Due to the change in alternative PBP2a, *β*-lactam antibiotics are not able to block this reaction [21,22]. Hovewer, ceftaroline, which belongs to *β*-lactam antibiotics, shows an activity even in MRSA strains [21]. This interesting fact was explained due to crystallographic studies of PBP2a, which, apart from the active site, indicated presence of an allosteric one in non-penicillin-binding domain [21,22]. Moreover, Otello et al. pointed out that an incorporation of a compound to allosteric site changed the active site into open conformation, which led to restoration of *β*-lactam antibiotics activity [21].

### 1.4. Potency of Imidazolones in Battle against Bacterial MDR

**Figure 1:**
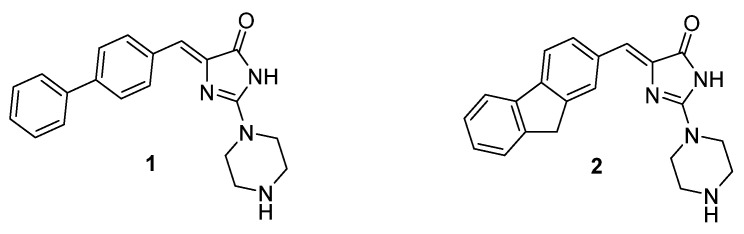
The previously found 5-arylideneimidazolones able to block AcrAB-TolC efflux pump (**1**, **2**) [23].

Notably, our previous studies proved that imidazolones and hydantoins displayed significant activities as antibiotic adjuvants in both, Gram-positive and Gram-negative, MDR bacteria. Arylideneimidazolones with unsubstituted piperazine at position 2 were highly active as EPIs of the AcrAB-TolC pump in MDR strains of *E. coli* (**1** and **2**, Figure 1) during the real-time efflux (RTE) assays. Based on results in microdilution assays, a presence of non-condensed rings, i.e., biphenyl, in comparison to condensed ones, i.e., fluorene (**1** vs. **2**, Figure 1), significantly affected abilities of molecules to increase efficacy of more diverse antibiotics. Compounds **1** and **2** were promising, although they displayed cytotoxicity in human liver carcinoma cells (HepG2) at concentrations required for adjuvant activity assays. These results pointed out a need for further modifications in the arylideneimidazolone group [23].

**Figure 2:**
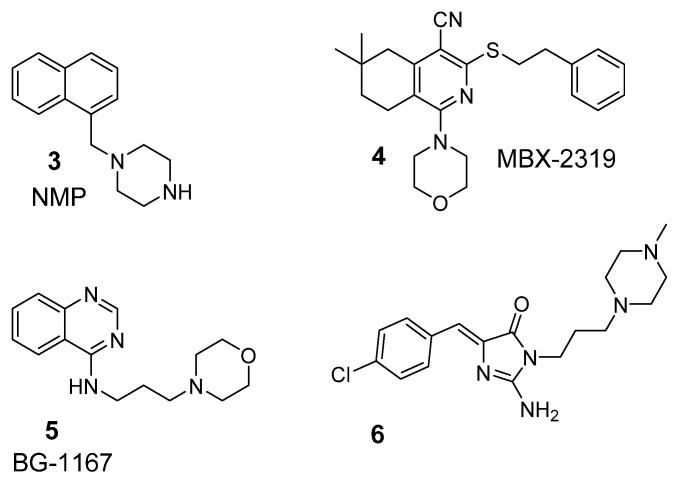
Previously identified antibiotic adjuvants containing: methylpiperazine i.e., NMP (**3**) or morpholine i.e., MBX 2319, BG1167 (**4** and **5,** respectively) as well as active oxacillin chemosensitizer from 5-arylideneimidazolones (**6**) [24,25,26,27].

Thus, next studies included modifications of either aromatic or amine fragments. The amine moieties were chosen on the basis of already described active adjuvants, which possessed either methylpiperazine, e.g., NMP (**3**, Figure 2) or morpholine fragment, e.g., MBX 2319 and BG1167 (**4** and **5**, Figure 2) [24,25,26]. Moreover, linker between the amine and imidazolone core was implemented. Crystallographic analysis for the chlorobenzylidene derivative (**6**, Figure 2) showed that Dimroth rearrangement surprisingly modified the substituents topology, and thus, methylpiperazine was connected by linker to the imidazolone core at position 3 [27]. The studies indicated an ability of the new imidazolones to block different MDR mechanisms in both, Gram positive and Gram negative, bacteria. They pointed out that two compounds possessing morpholine instead of the methylpiperazine moiety were even more potent oxacillin adjuvants in the MRSA strain investigated. In the case of MDR *Klebsiella aerogenes* (*Enterobacter aerogenes* in our previous papers), those series of compounds did not potentiate antibiotics effectiveness, but demonstrated EPI action in the real-time efflux (RTE) assays using the strain with overexpression of AcrAB-TolC efflux pump [27].

Those interesting results, but limited for only two morpholine-imidazolone representatives tested in only one MRSA strain (MRSA HEMSA 5), distinctly indicated a strong need of further extended studies in this chemical group, including both, wider chemical modifications and greater variety of MDR strains involving additional antibiotics. Furthermore, “drugability” questions should have been taken into account.

### 1.5. Research Purpose and Scope

**Figure 3:**
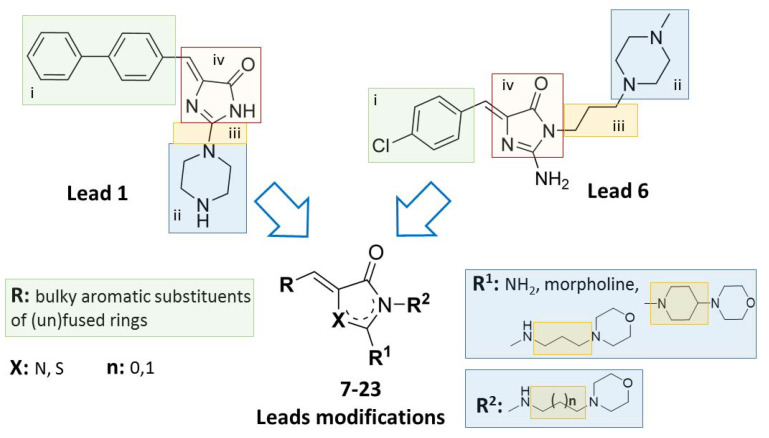
Lead structures (**1** and **6**) and their modifications performed within this study [23,27].

In response to the aforementioned challenges, and electing two previously found compounds (**1** and **6**, Figure 1 and Figure 2, respectively) as lead structures, we decided to complete a series of morpholine-imidazolones, including a few members found before, in order to investigate the role of topological variety within this chemical group for reversing bacterial MDR. For this purpose, the following modifications within respective areas of the lead structures (**1** and **6**) have been arranged: (i) the aromatic moiety at position 5, (ii) an exchange of piperazine (at position 2 or 3, respectively) into morpholine, (iii) modifications within the linker between the morpholine and imidazolone, (iv) an exchange of imidazolone core into thiazolone one (Figure 3).

Thus, a series of new 5-arylideneimidazolones (**7**–**21**, groups A and B) or 5-arylidenethiazolones (**22** and **23,** group C) containing the morpholine motive either at position 2 (group A and C) or at position 3 (group B), was investigated within this study (Table 1 and Table 2). Synthesis of new compounds and crystallographic studies for one representative (**7**) were performed. Antibiotic adjuvant properties for compounds **7**–**23** were evaluated in comprehensive microbiological assays, including tests in MDR Gram-positive and Gram-negative strains. Chosen compounds were tested in extended screening with a panel of antibiotics and various clinically derived *S. aureus* strains. In order to test their impact on Gram-negative bacteria, compounds were examined for their EPI activity in AcrAB-TolC expressing *K. aerogenes* in RTE. Next, molecular modelling studies were performed to determine probable mechanisms of the adjuvant action. For selected compounds, “druglikeness” was examined, i.e., water solubility as well as chemical and metabolic stability and potential drug-drug interactions (DDI) using both in silico and experimental assays. Finally, the structure-activity relationship (SAR) has been analyzed.

## 2. Results and Discussion

### 2.1. Synthesis

**Scheme 1:**
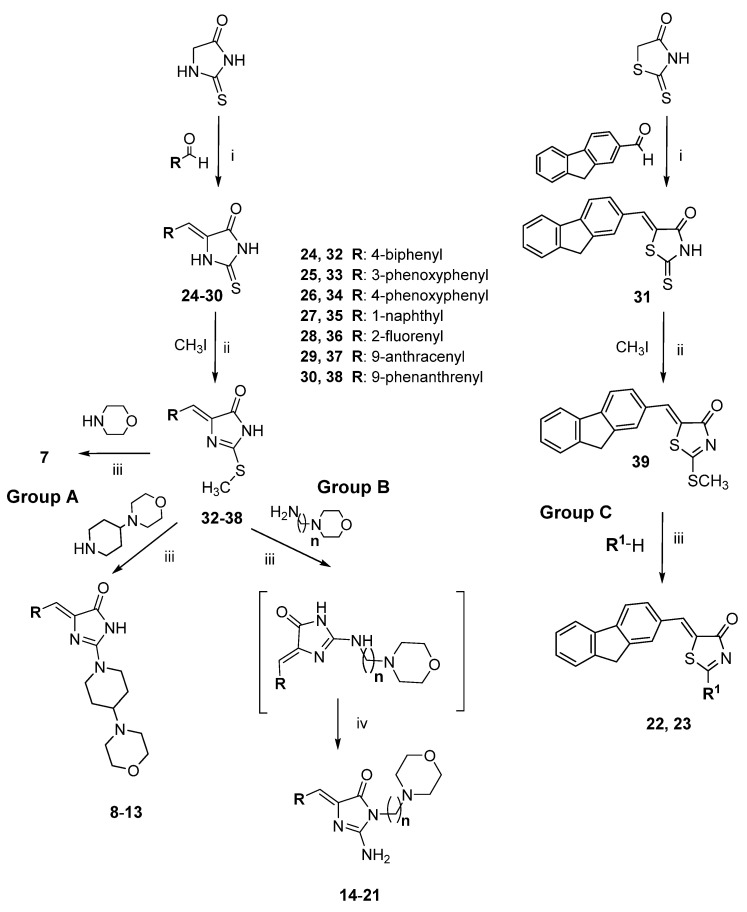
Route of synthesis for arylideneimidazolones (**7**–**21**; group A and B) and arylidenethiazolones (**22**, **23**; group C): (i) appropriate aldehyde, CH_3_COONa, CH_3_COOH, reflux, 5–7 h, then rt, 20 h; (ii) CH_3_I, EtONa, rt, 24 h; (iii) appropriate amine, 120–130 °C, 15 min, then EtOH, reflux, 5–6 h, then rt, 20 h; (iv) Dimroth rearrangement.

The routes for the synthesis of imidazolone (**7**–**21**) and thiazolone (**22**, **23**) derivatives are depicted in Scheme 1, while the synthesis of compounds **7**–**10**, **12**–**17, 19**–**21, 24**–**30, 32**–**38** has been described elsewhere [27,28]. In the first step, Knoevenagel condensation was performed, in which imidazolidinone or thiazolidinone reacted with an appropriate aromatic aldehyde to access the intermediates, namely 5-arylideneimidazolidinones (**24**–**30**, Scheme 1) or 5-arylidenethiazolidinone (**31**, Scheme 1). These compounds (**24**–**31**) were *S*-methylated to obtain further intermediates (**32**–**39**). Then, the *S*-methyl group at position 2 was replaced by morpholine-containing amine moieties (**7**–**13, 22** group A and C) or primary amine (**14**–**21, 23** group B and C) via the reaction between compounds **32**–**39** and commercially available primary or secondary amines. When the primary amines were employed in the reaction with imidazolone derivatives (**32**–**38**), Dimroth rearrangement was observed [27].

### 2.2. X-ray Crystallography

**Figure 4:**
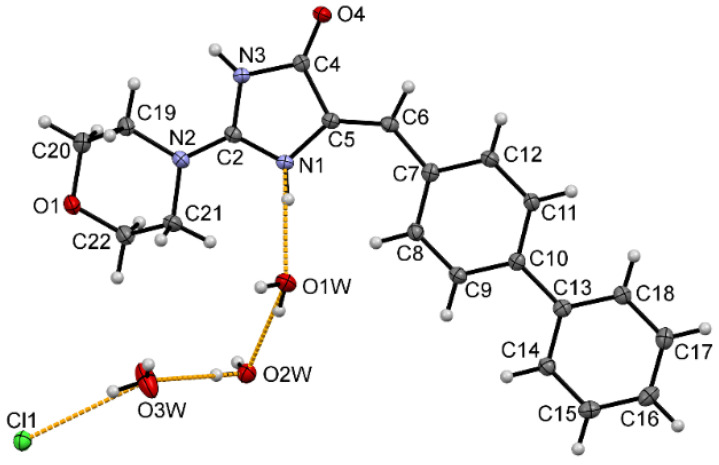
The molecular structure of protonated molecule **7** with the atom numbering scheme. Displacement ellipsoids are drawn at the 50% probability level. Orange dashed lines show hydrogen bonds between the chloride anion, three water molecules and protonated N1 atom.

**Figure 5:**
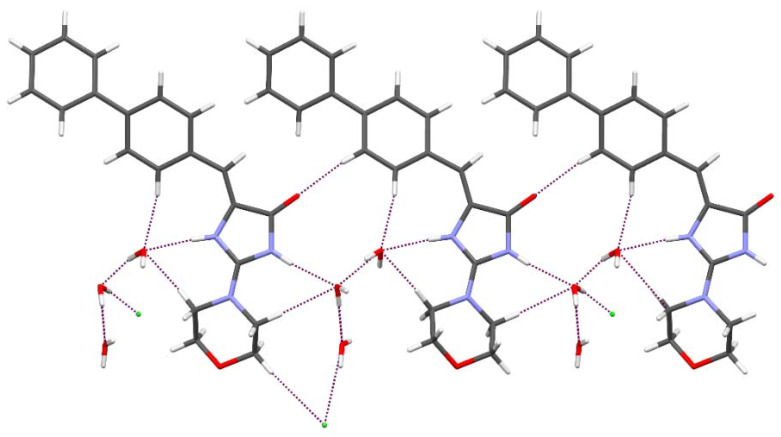
The intermolecular interactions of **7** in the crystal. Purple dashed lines indicate hydrogen bonds.

In order to determine the structural properties of this interesting group of morpholine-imidazolones, crystallographic analysis for compound **7** was performed. The molecular structure and atom numbering scheme are shown in Figure 4. In the crystal structure presented, the asymmetric unit consists of one cation being the protonated compound **7** at N1 atom, one chlorine anion and three water molecules. Molecule **7** possesses *Z* configuration at the C5=C6 double bond. We have observed the same atomic arrangement at the double bond also in other crystal structures of 5-arylideneimidazolone derivatives determined earlier [29,30]. The 5-arylideneimidazolone fragment is almost planar with a dihedral angle of 8.67° between both rings. The angle between the planes of the imidazolone and the second aromatic ring is 10.28°. The morpholine ring at C2 adopts chair conformation with the intermediate location of the substituent at N2 between equatorial and axial position. The torsion angle C2-N2-C19-C20 is 134.39°. The protonated N1 atom is engaged in a charge-assisted hydrogen bond with the water molecule O1W. It is worth noting that a search of the Cambridge Structural Database (CSD) [31] for the protonated 5-arylideneimidazolone ring containing a N atom at C2 atom resulted only in one crystal structure [32]. The hydrogen bonding pattern presented and involving a protonated hydrogen atom, three water molecules and chlorine anion is very seldom observed in the CSD [31]. The packing of molecules in the crystal is dominated by N-H···O, O-H···Cl, O-H···O and C-H···O intermolecular hydrogen bonds (Figure 5), whose parameters are listed in Table 3.

### 2.3. Biological Screening in S. aureus

In terms to assess the potential adjuvant activity of compounds (**7**–**23**) in Gram-positive bacteria, microbiological assays with *S. aureus* strains were performed. In these studies, different MSSA, MRSA and Vancomycin-intermediate *S. aureus* (VISA) were used. The adjuvant effect of compounds was evaluated in combination with several antibiotics, i.e., oxacillin, ampicillin, vancomycin, ciprofloxacin and erythromycin. Compounds (**7**–**23**) were examined in combination with oxacillin in MSSA isolate ATCC 25923 and the MDR isolate MRSA 19449. Selected 5-arylideneimidazolones (**14**–**16**) were investigated additionally with a panel of antibiotics in two MSSA and eight MRSA strains. Moreover, potential mechanisms of action for the series were determined employing docking and molecular dynamics simulation with both, active and allosteric, sites of PBP2a.

#### 2.3.1. Antibacterial Activity in *S. aureus*

The intrinsic antibacterial activity of the seventeen 5-arylideneimidazolone derivatives was assessed by determining their minimal inhibitory concentration (MIC) values in ATCC 25923 and MRSA 19449. Results are presented in Supplementary (Table S1). The results indicated that none of 5-arylideneimidazolones analyzed displayed a therapeutically relevant stand-alone antistaphylococcal activity. The MIC values of compounds **7**–**17, 19**–**23** were in the range of 0.03125 – 0.25 mM against both *S. aureus* strains with the exception of compound **18**, which displayed even higher MICs of 1 mM and 2 mM in ATCC 25923 and MRSA 19449, respectively. Results for compounds **16** and **20** in ATCC 25923 strain were published previously [27]. These findings suggest that the compounds are not likely to become antibiotics by themselves, and thus are unlikely to elicit antibiotic resistance formation when added as an adjuvant molecule. Nonetheless, for a number of compounds, precipitation was observed after the addition of bacterial suspension in MH II broth (compounds **8**, **10**, **15**–**18**, and **22**), and thus, reported MICs for these compounds should be interpreted with caution.

#### 2.3.2. Adjuvant Effects for Oxacillin

Given the lack of a clear antistaphylococcal activity of any of the 5-arylideneimidazolones and 5-arylidenethiazolones tested here, we next tested their chemosensitizing capabilities in combination with the penicillinase-resistant *β*-lactam antibiotic oxacillin. For this purpose, compounds **7**–**23** at a concentration of 1/4 of their MICs were added to two-fold serial dilutions of oxacillin. Moreover, the effectiveness of the antibiotic alone was tested, which yielded MICs of 0.25 µg/mL for ATCC 25923 and 256 µg/mL for MRSA 19449. Potentiating effects of compounds were then determined by comparison of the efficacy of oxacillin tested in the presence and absence of compounds and expressed as activity gain A (Table 4). Detailed results for the whole series are shown in Supplementary (Table S2). Among the series of compounds tested, two derivatives, compounds **10** and **15**, significantly enhanced the efficacy of oxacillin against MRSA strain 19449 (Table 4, Supplementary Table S2). When supplemented at a concentration of 0.0625 mM, compound **10** reduced oxacillin resistance of MRSA 19449 by four- to eight-fold. A slightly lower activity gain was determined for compound **15**, which once added at a concentration of 0.0625 mM, decreased the oxacillin MIC of MRSA 19449 by two- to four-fold. None of compounds tested here had any influence on the oxacillin MIC in the reference MSSA strain ATCC 25923.

#### 2.3.3. Extended Biological Screening in *S. aureus*

##### Antibacterial Activity in Additional Strains of *S. aureus*

Compounds **14**–**16** were tested further for their adjuvant-like effect with a set of ten additional *S. aureus* isolates. Firstly, MIC values of the molecules were evaluated in two clinical MSSA isolates (MM-O058, MM-N072), seven MRSA (USA300 LAC, 5328, LG-N017, MM-O021, R45-CC45, R46-CC22, COL) and one VISA strain (Mu50). Detailed results are presented in the Supplementary section (Table S3). In line with our observations described above, none of the 5-arylideneimidazolones **14**–**16** displayed any considerable antistaphylococcal activity against this set of *S. aureus* strains (MIC from 0.125 to ≥ 1 mM).

##### Ability to Enhance Antibiotic Activity

**Figure 6:**
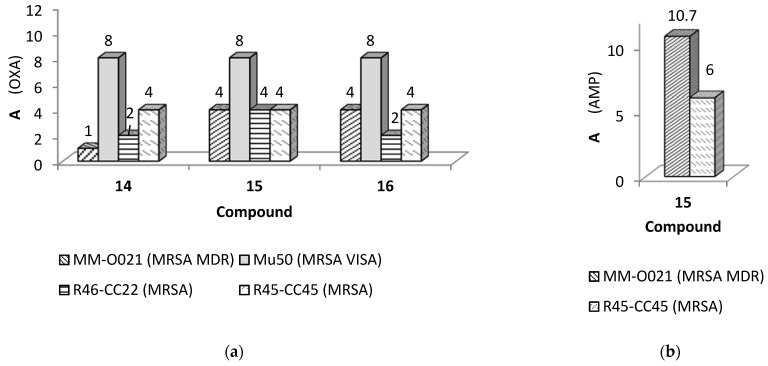
The gain of activity (A) for *β*-lactam antibiotics caused by active 5-arylideneimidazolone derivatives (**14**–**16**) in various clinical isolates of *S. aureus.* Antibiotics and clinical isolates of *S. aureus* used: (**a**) oxacillin (OXA) in strains: MM-O021, Mu50, R46-CC22 and R45-CC45, respectively; (**b**) ampicillin (AMP) in the strains: MM-O021 and R45-CC45. Assays performed in 3–4 repetitions; A, calucated according to Equation (1) (Section 3.3.1), shown as arithmetic mean in case of different values obtained in the repetitions; A ≥ 4 for active compounds potentiating effects of antibiotics. Detailed results for all strains explored are presented in Supplementary material (pp. 11–15).

The adjuvant properties of compounds **14**–**16** in *S. aureus* clinical isolates were next tested in combination with oxacillin and the penicillinase-susceptible *β*-lactam antibiotic, ampicillin. The second *β*-lactam antibiotic was chosen based on its vulnerability to enzymatic hydrolysis, another important resistance mechanism in *S. aureus* against penicillin. Therefore, the results of the MIC reduction assay obtained on the activity of arylideneimidazolones combined with oxacillin and ampicillin, respectively, might provide further insights into the possible mechanism of action of the compounds. Complete results are presented in Supplementary (Tables S4 and S5), while condensed in Figure 6, in the case of desired activity noted. The results obtained in this set of experiments indicated that each of the compounds tested at the concentration of 0.0625–0.125 mM had the capacity to reduce significantly the resistance to oxacillin of some but not each of the MRSA strains tested (Figure 6a, Supplementary Tables S4 and S5). One possible explanation for the lack of adjuvant activity of compounds in some of the strains selected for this study might be that these strains express PBP2a only when induced by the antibiotic, while others, for which potentiating activity of compounds was observed, produce this protein in high levels straight away.

Compounds **14**–**16** were able to decrease MIC values of oxacillin by four- to eight-fold in MRSA isolates MM-O021, RR45-CC45, and RR46-CC22, and the VISA isolate Mu50 (Figure 6a). Notably, compound **15** was also able to decrease the MIC values for ampicillin in the MRSA strains MM-O021 and R45-CC45 (Figure 6b), with A-values 10.7 and 6, respectively, while the ampicillin MICs of the other tested strains were not markedly affected by compounds **14**–**16** (Supplementary Tables S6–S9). Similarly, these compounds neither influenced the activity of oxacillin in MSSA strains MM-O058 and MM-N072, nor altered the oxacillin MICs of MRSA strains USA300 LAC, 5328, LG-N017, and COL.

To evaluate whether compounds **14**–**16** might affect the MICs of the set of strains investigated for other antibiotic classes, we next tested the chemosensitizing properties of compounds **14**–**16** in combination with the glycopeptide vancomycin, the fluoroquinolone ciprofloxacin, and the macrolide erythromycin. None of derivatives altered the MICs for vancomycin, ciprofloxacin, and erythromycin in these strains of *S. aureus* (Supplementary Tables S10–S14), suggesting that the compounds interact specifically with PBP2a in some but not each strain of MRSA.

#### 2.3.4. Molecular Modelling of the Interactions with PBP2a

**Figure 7:**
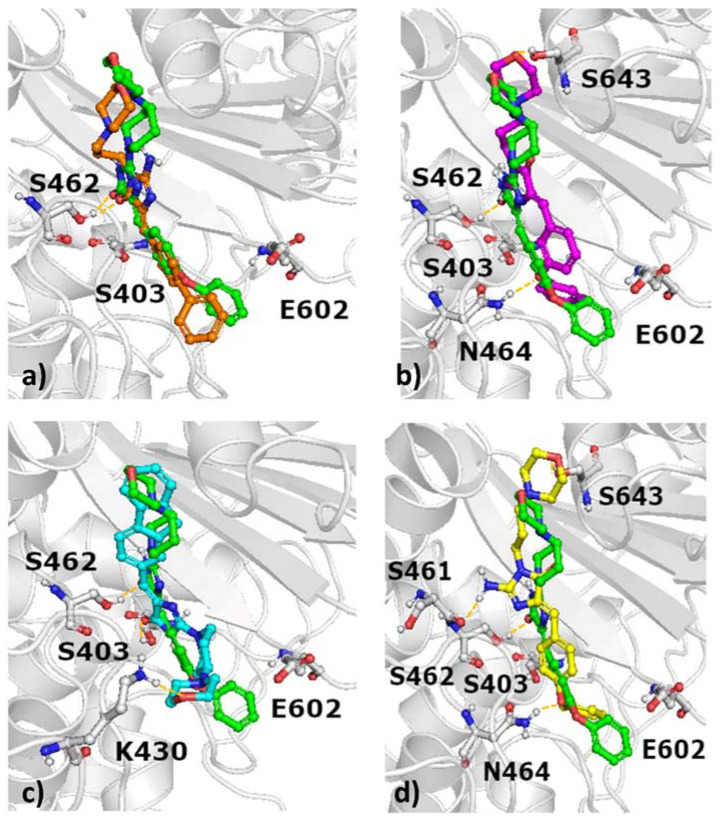
Docking results of selected compounds to the active site of PBP2a with indication of hydrogen bonds (**a**) green: **10**, orange: **15**; (**b**) green: **10**, magenta: **16**, (**c**) green: **10**, cyan: **7**, (**d**) green: **10**, yellow: **9**.

**Figure 8:**
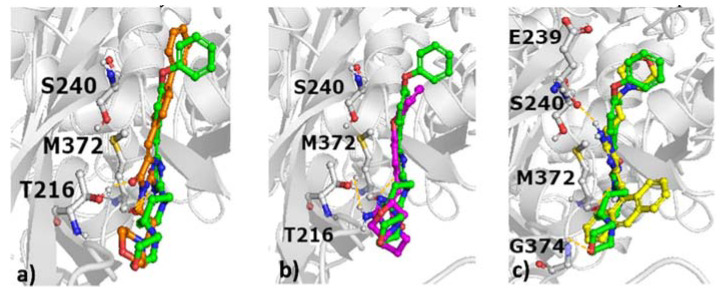
Docking results of selected compounds to the allosteric site of PBP2a with indication of hydrogen bonds; (**a**) green: **10**, orange: **15,** (**b**) green: **10**, magenta**: 9**, (**c**) green: **10**, yellow: **20**.

In order to test whether the activities of the compounds on the oxacillin MICs of MRSA are related to their interactions with PBP2a, we next performed a series of molecular modelling studies. The docking results of selected compounds to the PBP2a active site are presented in Figure 7, whereas docking to the allosteric site of PBP2a is shown in Figure 8 (complete data are available in the Supplementary Figures S1 and S2).

These modelling studies show that the docking poses of compounds to the active site are very similar (Figure 7), and there is no obvious difference between the orientation of compounds reducing the antibiotic MIC of oxacillin and compounds without this property.

Interestingly, when the compounds were modelled with the allosteric site of PBP2a, clear variations in orientations were noticed (Figure 8), although no consistent pose could be defined which may allow to discriminate between active and inactive compounds.

**Figure 9:**
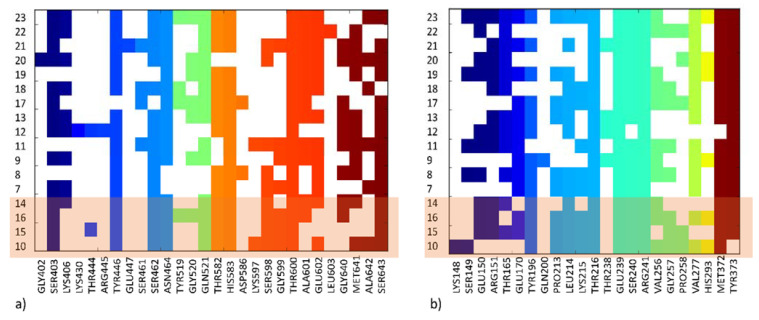
Ligand-protein interaction matrix depicting contacts in (**a**) the active site of PBP2a, (**b**) the allosteric site of PBP2a; compounds for which antibiotic adjuvant properties have been indicated in our investigations are shaded.

To facilitate the interpretation of the results, ligand-protein interaction matrices were prepared (Figure 9), which captured the various interactions occurring in ligand-PBP2a complexes as defined in docking.

This interaction matrices highlight differences in the set of interactions formed by particular structures, both at the active (Figure 9a) and allosteric sites (Figure 9b) of PBP2a. Compounds with antibiotic adjuvant properties (Figure 7) share similar positions of the compound core in the PBP2a active site, although they adopt different orientations of the aromatic moieties, with varying distances and angles towards Glu602. The position of the most active compound **10** is in the middle between the less active **15** depicted in orange (Figure 7a) and **16** depicted in magenta (Figure 7b). Both **10** and **15** form hydrogen bond with S462, whereas **16** interacted via hydrogen bond with N464. Compound **7** (Figure 7c), which is characterized by a different chemical structure also adopt different orientation in the active site of PBP2a. It is also indicated by the set of hydrogen bonds formed (with S403, K430). Compound **9** adopt similar docking pose in the PBP2a active site to **16** (Figure 7d and Figure 7b, respectively); however, when docking to the PBP2a allosteric site is considered, **9** is the only compound which makes contact with Gln200 and the only one not in contact with any of the amino acids from the set Lys148, Ser149, Glu150, and Arg151 (Figure 8b, Figure 9). Further analysis of docking outcome to the allosteric site of PBP2a revealed that the most active compound **10** orients its aromatic ring perpendicular in comparison to other active compounds (e.g., **15**, Figure 8a), and as the only compound has contact with Lys148. Compound **10** is also the only compound which does not interact with Tyr373 (Figure 9). The majority of compounds also form hydrogen bond with M372 and T216, whereas **20** is bonded via this type of contact to E239, and G374.

**Figure 10:**
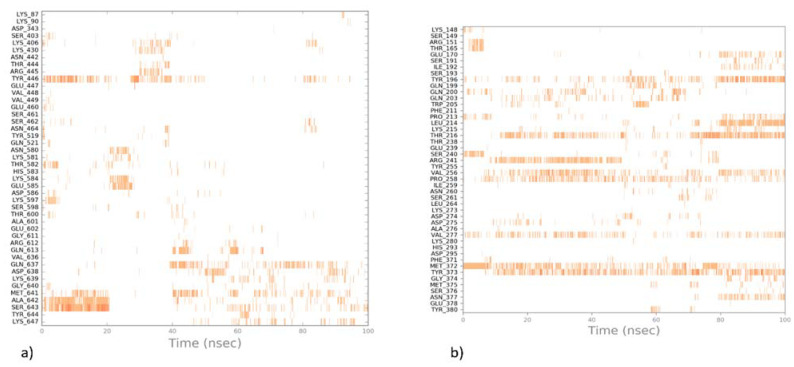
Ligand-protein interaction diagrams obtained by molecular dynamic simulations for the most active compound **10** for (**a**) the active site; (**b**) the allosteric site of PBP2a.

As docking captures only one moment of ligand-protein contact, molecular dynamic simulations were performed to provide a more detailed picture of the possible mechanism of action of active compounds with PBP2a. Figure 10 presents results for the most active compound **10**, whereas results for compounds **14**–**16** are presented in the Supporting Information (Supplementary Figures S3 and S4).

Molecular dynamic simulations show that the stability of different poses of compounds in the allosteric site is much higher than in the active site of PBP2a, as the compound moves from its initial position and does not adopt any stable position in the active site. This suggests that the compounds studied most probably act as allosteric agents, which may improve the ligand-protein contacts in the active site.

### 2.4. Biological Screening in the Gram-Negative Bacterial Species K. aerogenes

#### 2.4.1. Chemosensitizer Activity in *K. aerogenes*

The influence of 5-arylideneimidazolones (**7**–**15, 17**–**19, 21**) and 5-arylidenethiazolones (**22**, **23**) was also tested with *K. aerogenes* strain Ea-289, which overexpresses the AcrAB-TolC efflux pump and exhibits a porin-deficient phenotype [33]. At first, MIC values of compounds were determined (Table S15, Supplementary). Next, their abilities to reduce MICs of the AcrAB-TolC substrates doxycycline (a tetracycline-class antibiotic) and erythromycin were studied (Table S16, Supplementary). These studies indicated that the addition of the arylidene compounds did neither affect the viability of strain Ea-289, nor caused significant reductions in the MIC values associated with doxycycline and erythromycin.

A subset of compounds, namely **7**, **12**–**16**, **19**, **20**, was also examined for the potential to increase the effectiveness of the antibiotics chloramphenicol, erythromycin, doxycycline, and the fluoroquinolone antibiotic norfloxacin, which all are substrates for AcrAB-TolC in *K. aerogenes* [34,35]. In addition to strain Ea-289, the adjuvant-like effect of the compounds was investigated in strain CM-64, which also overexpresses the AcrAB-TolC efflux pump, although it has no changes in porin content [36], and in the Ea-289 derivatives, i.e., Ea-294 and Ea-308, which are devoid of AcrAB (Table S17, Supplementary). Similar to the situation in strain Ea-289, none of the compounds displayed a clear antibacterial activity against the set of *K. aerogenes* strains (Table 5), although compounds **15**, **16**, and **19** were more effective (≥ 4-fold) in strains Ea-294 and Ea-298 when compared to the parental strain Ea-289, suggesting that **19** might interact with AcrAB-TolC. In contrast, the lack of an apparent difference in MICs between the AcrAB-TolC over-producing Ea-289 and the parental strain observed for compounds **7**, **12**, **13**, **14**, and **20** suggests that these compounds do not interact with this multidrug efflux pump, although such an interaction cannot be excluded since the real MIC values for these compounds in Ea-289 are much higher than 2 mM, the highest concentration for compounds tested in our assays.

Additionally, none of the compounds **7**, **12**–**16**, **19**, or **20** were able to reduce MICs of the antibiotics (A < 2), when added at concentrations corresponding to 1/4 of their MIC, which is in line with earlier findings for 5-arylideneimidazolones and *K. aerogenes* [27]. The results for compounds **16** and **20** obtained in the MIC reduction assay were described previously [27] or are presented in the Supplementary section (Supplementary Table S17).

#### 2.4.2. Real-Time Efflux Assay

Compounds **7**, **12**–**16**, **19**, and **20** were also assessed for their EPI property in *K. aerogenes* strain Ea-289 with the use of a real-time efflux (RTE) assay. Results for compounds **16** and **20** were previously described [27]. The experiment is based on the measurement of the extrusion of a fluorescent dye from bacterial cells overproducing efflux pump transporters in the presence and absence of compounds tested. The dye used in this assay, 1,2’ –dinaphthylamine (1,2’-dNA), is a substrate for the AcrAB-TolC efflux pump, and is strongly fluorescent upon incorporation into a phospholipid bilayer, although it is almost non-fluorescent in an aqueous solution [37,38].

**Figure 11:**
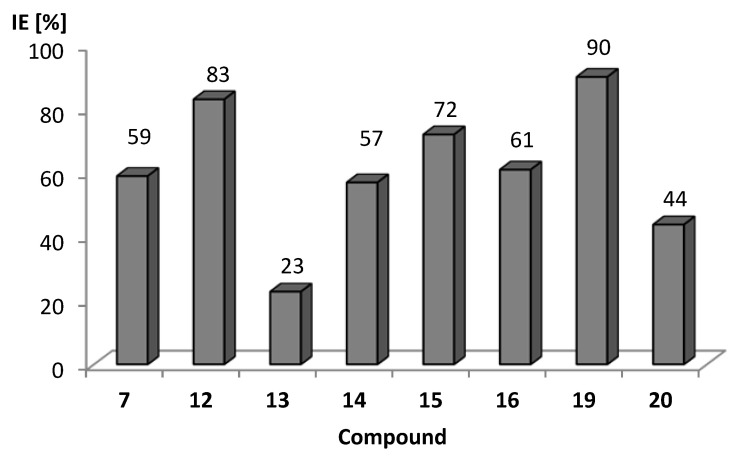
Inhibition efficiency (IE) of imidazolone derivatives **7**, **12**–**16**, **19**, and **20** towards extrusion of 1,2’-dNA in *K. aerogenes* strain Ea-289 over-producing AcrAB-TolC. IE calculated according to Equation (2) (Section 3.3.2.). IE=100% if a tested compound totally inhibits the dye-substrate efflux.

The data obtained within this assay indicated that the addition of the 5-arylideneimidazolones led to a decrease in the fluorescence intensity of 1,2’-dNA in bacterial cells treated with these compounds. As our previous study already indicated an increased fluorescence quenching due to molecular interactions between the fluorophore and 5-arylideneimidazolones [27], we quantitatively compared the effectiveness of 5-arylideneimidazolones in the inhibition of the extrusion of 1,2’-dNA by calculating the inhibition efficiency (IE) of each compound. This comparison showed that the compounds tested were able to inhibit the extrusion of the fluorescent dye out of *K. aerogenes* Ea-289 cells (Figure 11). The highest EPI activity was observed for compound **19**, which blocked the export of 1,2’-dNA in Ea-289 by 90 %. A likewise strong blocking efficacy (IE = 83 %) was also noticed for compound **12**, while the lowest inhibitory effect was found for compound **13** (IE = 23 %).

### 2.5. Drug-Likeness Study

#### 2.5.1. Water Solubility

Six representatives of 5-arylideneimidazolones (**9**–**12, 15, 19**) were chosen for the determination of water solubility. UV spectroscopy was used for the estimation of concentrations of compounds according to the method described earlier [39,40]. Acquired UV absorbance, calculated means, and standard deviations for tested compounds are presented in Supplementary Table S18. Solubility results, presented in Table 6, point out that compounds from the same group (A or B, Table 1 and Table 2) with the two-ring aromatic moiety are more soluble than compounds with the fluorene motive (Group A: **9**, **10**, **11** vs. **12**; Group B: **15** vs. **19**). Moreover, comparing compounds with the same arylidene moiety (**12** vs. **19**) and various positions of amine motives, such as amine at position 2 or 2 and 3, respectively, show that the presence of a morpholine moiety connected by a propyl linker at position 3 (**19**) improves the water solubility (from 1.645 to 19.467 mM/L). The most soluble of tested compounds was compound **15**, which could be dissolved in water at a concentration of up to 205.502 mM/L.

#### 2.5.2. Stability in Different pH Conditions

Compounds **9**–**12**, **15**, and **19** were also tested for their stability in acidic and basic conditions. This assay was carried out for 48 h and samples were taken at six-time intervals (10 min, 1 h, 2 h, 3 h, 24 h, 48 h). Stability of the compounds was determined using the TLC method. These studies confirmed that 5-arylideneimidazolones are usually more stable in acidic conditions, with four out of six compounds (**10**–**12**, **19**) being stable for at least 2 h, and compounds **10**–**12** being stable for 24 h. In basic conditions, only compounds **10** and **15** were stable for 24 h. Compound **10** was the most stable one**,** giving one degradation product which was observed, in both conditions, after 48 h. Results with details, including time of degradation of products obtained, are presented in the Supplementary (Tables S19 and S20).

#### 2.5.3. Drug-Drug Interactions (DDI)

Considering that the compounds are designed to be used clinically in combination with antibiotics, information about potential DDI is crucial. For this reason, metabolic in silico and in vitro assays were performed. In computer-aided studies, the MetaSite program was used to predict potential products of CYP3A4 metabolism for the entire series due to a significant involvement of this isoform of cytochrome P450 in drug metabolism. In case of in vitro assays, establishment of IC_50_ of CYP3A4 was performed for the most active compounds **15** and **19** selected on the basis of the biological screening.

##### In Silico Assays

Possible metabolic paths and sites of metabolic biotransformation were predicted using the MetaSite 6.0.1 computer program provided by Molecular Discovery Ltd. The probable sites of metabolism were obtained with an application of computational models of liver, skin, brain and cytochromes. In the majority models, the most probable site of metabolism for the compounds tested are positions 3 and 5 in the morpholine ring. In the case of CYP3A4 metabolism, the most likely places of modification dependent on the position of amine, i.e., only positions 3 and 5 in morpholine ring were more than 60% probable for the amine at position 2 (**7**–**13**, **22**, **23**), whereas the alkyl group connected directly to morpholine was also significantly probable for compounds with the aminealkyl fragment at position 3 (**14**–**21**). The most probable mechanism in CYP3A4 metabolism was *O*-dealkylation. Additionally, *N*-dealkylation (**14**–**21, 23**), dehydrogenation (**7** and **14**) and aliphatic carbonylation (**14**–**21**) were the highly probable (>60%) mechanisms. Results for compounds **15** and **19**, selected for in vitro assays, are presented in the Supplementary (Figures S5 and S6, respectively).

##### DDI In Vitro Assays

**Figure 12:**
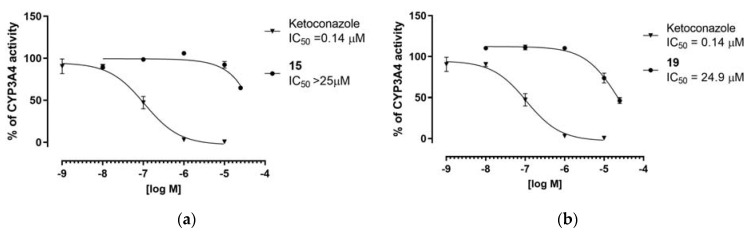
Inhibition of CYP34A activity by compounds **15** (**a**) and **19** (**b**) and reference ketoconazole experssed with IC_50_ values.

The most active chemosensitizers found, either for Gram-positive (**15**) or Gram-negative (**19**) strains, were tested for their DDI-risk using the luminescent CYP3A4 P450-Glo™ assay. Ketoconazole served as a reference inhibitor of CYP3A4. Although predicted in silico as showing a high probability of morpholine ring interaction with CYP3A4, compounds **15** and **19** did not influence CYP3A4 in vitro in concentrations lower than 25 µM (Figure 12). Even at the highest concentration tested, 25 µM, compound **15** did not reach 50% of inhibition of CYP3A4 (Figure 12a). Similarly, compound **19** was a weak CYP3A4 inhibitor with IC_50_ = 24.9 µM (Figure 12b).

Results obtained in this study indicated favorable “drug-like” properties for the compounds tested, notably a low risk of potential DDI. Compound **15**, in particular, did not affect CYP3A4 within the concentration range tested.

### 2.6. Structure Activity Relationships

5-Arylideneimidazolones (**7**–**21**) and 5-arylidenethiazolones (**22**, **23**) possess an amphiphilic structure. They include a bulky aromatic moiety at position 5 and an amine fragment at position 2 (group A and C, Table 1 and Table 2) or at position 2 and 3 (group B, Table 2), containing a differently linked morpholine. This variety of topology, together with biological screening results, enables a comprehensive analysis of possible structure-activity relationships (SAR).

Compounds (**7**–**23**) were tested for their ability to restore oxacillin activity in MRSA 19449, and two of them (**10** and **15**) showed significant (4 – 8-fold) reduction of the antibiotic MIC (Table 4). Additionally, the assays performed in ten *S. aureus* strains (2 MSSA, 7 MRSA and 1 VISA) for compounds **14**–**16** displayed that these compounds were able to decrease oxacillin MIC up to 8-fold (Figure 6a). Notably, the active oxacillin “adjuvants” (**10**, **14**–**16**) therefore have been identified among imidazolones, and three of them represent Dimroth-rearranged topology (**14**–**16**). It is worth to underline that the arylidene moiety of the active compounds is limited to only non-condensed rings, i.e., the phenoxyphenyl (**10**, **16**) or biphenyl (**14**, **15**) moieties. This is in contrast to previous studies, which have indicated the fused rings as distinctly more profitable in the case of imidazolones with the corresponding amine topology (position 3) [27,41]. In accordance with the present results, the preceding studies have also shown that amine at position 2 and 3, corresponding to group B, is more profitable, although different amines have been under consideration [27]. This suggests a predominant role for the substitution topology of the compounds obtained by Dimroth rearrangement. For instance, morpholine looks as it is a good bioisoster of the methylpiperazine presented in the previously found oxacillin “adjuvants” [27]. A deeper insight into structural features of the active compounds (**10** and **14**–**16**) demonstrates the aromatic biphenyl moiety as the most favorable (**15** vs. **16**). Furthermore, the linker between the morpholine and the imidazolone has some impact, i.e., the longer (C3) linker could be superior compared to the shorter (C2) one (**15** vs. **14**, Table 4, Supplementary Table S2 and Figure S6). The assays with ampicillin in the same strains, performed for compounds **14**–**16**, reinforce this conclusion. Hence, the biphenyl-derived C3-linked imidazolone compound **15** was outstanding among these three, causing significant reduction of the ampicillin MIC in two MRSA strains (Figure 6b).

The longer linker (C3) is associated with better flexibility and this could explain the different orientations of the aromatic moieties of the compounds adopting to the allosteric site of PBP2a. Following the example of the linker of the group B, this should also apply to chemical functions less rigid than ones of groups A and C, although this conclusion requires further studies based on a larger population of compounds.

Summing up, results obtained in various MRSA strains point out the beneficial role of (1) non-fused aromatic rings, i.e., biphenyl and phenoxyphenyl ones, (2) Dimroth-rearranged topology of substituents at the imidazolone core (group B) and (3) C3-linker for the cyclic amine at position 3 of the imidazolone, for the desirable adjuvant activity. Taking into consideration the other modifications performed, it looks as if the substitution with a morpholine-containing amine at position 2 (group A) was less effective, while the replacement of the imidazolone with the thiazolone (group C) failed.

Furthermore, compounds **7**–**23** were tested for their chemosensitizer activity in the MDR strain of *K. aerogenes*. None of these compounds showed significant reduction of either doxycycline or erythromycin MIC. Nevertheless, RTE assays indicated that these compounds were able to block the dye-substrate efflux by AcrAB-TolC pump, including six members (**7**, **12**, **14**–**16**, **19**) causing potent inhibitory action (IE >50 %). Two the most active compounds, namely **12** and **19**, contain the triple fused ring of fluorene. Interestingly, another triple-fused ring moiety, i.e., anthracene, provided significantly lower EPI potency compared to compounds with the same amines and similar substituent topology (**19** vs. **20** and **12** vs. **13**, Figure 11). In general, the role of the aromatic substituent at position 5 should be crucial for the EPI properties to give the following order: fluorene (**19**, **12**) > phenylbenzene (**15**, **7**, **14**) > phenoxybenzene (**16**) > anthracene (**20**, **13**). An impact of the substituent topology has also been noted, indicating the beneficial role for the Dimroth-rearranged topology of group B in comparison to the 2-extended topology of group A (**19** vs. **12**, **15** vs. **7** and **20** vs. **13**, Figure 11). In the case of compounds with different lengths of linker (C3 vs. C2), the longer linker provided a distinctly more potent AcrAB-TolC inhibitory action, as noticed in **15** vs. **14**, Figure 11. Thus, the last two SAR conclusions for *K. aerogenes* are in good agreement with those from the studies with *S. aureus*. Although the fluorene derivatives **12**, **19**, the most active AcrAB-TolC EPI in this study, did not act as oxacillin adjuvants in *S. aureus*, a similar EPI activity was found for the active adjuvants **14**–**16**. The biphenyl compound (**15**), which was identified as the most active in the various *S. aureus* assays, especially, provides an interesting example in the search of an antibiotic adjuvant with a wide range of actions, including MDR mechanisms of both Gram-positive and Gram-negative bacteria.

Results for compounds **9**–**12**, **15**, **19**, investigated on their drug-like properties, indicated that the most promising *p*-phenylbenzylidene derivative **15**, demonstrated also the most favorable water solubility (205.502 mM/L) among the compounds studied. In the chemical stability assay conducted at various pH values, the best stability was obtained for the 4-phenoxybenzylidene compound (**10**)**,** while compound **15** stood out in this series as more stable under alkaline condition than under acidic ones.

The assays in vitro on CYP3A4 inhibitory properties for the most active compounds from the studies with *S. aureus* (**15**) and *K. aerogenes* (**19**) indicated that the fluorene analogue (**19**) had slightly less favorable properties than those of **15**, showing a weak CYP3A4 inhibition (IC_50_=24.9 µM, Figure 12b). Hence, the *p*-phenylbenzylidene derivative **15** gives a good prospect to be combined with antibiotics which are metabolized through CYP3A4, due to rather negligible risk of effects on the metabolism of these drugs.

## 3. Materials and Methods

### 3.1. Chemical Synthesis

Reagents were purchased from Sigma Aldrich (Darmstadt, Germany), Alfa Aesar (Karlsruhe, Germany) or Acros Organics (Geel, Belgium). The progress of the reactions was monitored by thin layer chromatography (TLC). It was carried out on silica gel 60 F254 plates (0.2 mm Merck, Darmstadt, Germany). UV light was used for the visualization of spots. A MEL-TEMP II apparatus (LD Inc., Long Beach, CA, USA) was employed to determine melting points (m.p.) and these mp are uncorrected. The ^1^H-NMR and ^13^C-NMR spectra were obtained on a Mercury-VX 300 Mz spectrometer (Varian, Palo Alto, CA, USA) in DMSO-d*_6_*. In ^1^H-NMR spectra, chemical shifts were reported in parts per million (ppm) on the δ scale. The solvent signal served as an internal standard. Data are reported as follows: Chemical shift, multiplicity (s, singlet; br.s, broad singlet; d, doublet; d def, doublet deformed; m, multiplet), number of protons, position of protons (Ar- aromatic moiety at position 5, Pp—piperidine, Mor—morpholine). Mass spectra were recorded—on a UPLC-MS/MS system consisting of a Waters ACQUITY^®^UPLC^®^ (Waters Corporation, Milford, MA, USA), which is coupled to a Waters TQD mass spectrometer (electrospray ionization mode ESI-tandem quadrupole). Chromatographic separations were carried out using the Acquity UPLC BEH (bridged ethyl hybrid) C18 column; 2.1 × 100 mm^2^, and 1.7 µm particle size, equipped with Acquity UPLC BEH C18 VanGuard precolumn (Waters Corporation, Milford, MA, USA); 2.1 × 5 mm^2^, and 1.7 µm particle size. The column was maintained at 40 °C and eluted under gradient conditions from 95% to 0% of eluent A over 10 min at a flow rate of 0.3 mL·min^−1^. Eluent A: water/formic acid (0.1%, *v*/*v*); eluent B: acetonitrile/formic acid (0.1%, *v*/*v*). Chromatograms were obtained using Waters eλ PDA detector. Spectra were analyzed in the 200–700 nm range with 1.2 nm resolution and sampling rate 20 points/s. The MS detection settings of the Waters TQD mass spectrometer were as follows: source temperature 150 °C, desolvation temperature 350 °C desolvation gas flow rate 600 L·h^−1^, cone gas flow 100 L·h^−1^, capillary potential 3.00 kV, cone potential 40 V. Nitrogen was used for both nebulizing and as drying gas. Data was collected in a scan mode ranging from 50 to 1000 *m/z* in time 0.5 s intervals. Data acquisition software was MassLynx V 4.1 (Waters Corporation, Milford, MA, USA). Retention times (t_R_) are given in min. The UPLC/MS purity of compounds **7**–**23** was determined (%). The synthesis of intermediates **24**–**30, 32**–**38** and products **7**–**10, 12**–**17, 19**–**21** was described earlier [27,28].

#### 3.1.1. General Procedure to Obtain 5-Arylidenethioxothiazolidin-4-one (**31**)

To flat-bottom flask 2-thioxothiazolidin-4-one (3.33 g, 25 mmol), acetic acid (25 mL), sodium acetate (8.33 g, 100 mmol) and appropriate aldehyde (25 mmol) were added. The reaction mixture was refluxed for 5 h. Then, it was stirred at room temperature for 20 h. The progress of the reaction was monitored by TLC (chloroform: ethyl acetate/1:1). Purification was performed using crystallization from acetic acid.

##### (*Z*)-5-((9*H*-Fluoren-2-yl)Methylene)-2-Thioxothiazolidin-4-one (**31**)

9*H*-Fluorene-2-carbaldehyde (25 mmol, 4.86 g) and 2-thioxothiazolidin-4-one (25 mmol, 3.33 g) were used. Yellow solid. Yield 63.73%; mp 259–261 °C. C_17_H_11_NOS_2_ MW 309.41. LC/MS±: (ESI) *m/z* [M + H]^+^ 310.16. ^1^H-NMR (DMSO-d_6_, ppm): δ 13.79 (s, 1H, N3-H), 8.05–8.00 (d def., 1H, Ar-5-H), 7.97–7.92 (d def., 1H, Ar-4-H), 7.75 (s, 1H, Ar-1-H), 7.67 (s, 1H, C=CH), 7.62–7.57 (m, 2H, Ar-3,8-H), 7.41–7.33 (m, 2H, Ar-6,7-H), 3.98 (s, 2H, Ar-9-CH_2_).

#### 3.1.2. General Procedure to Obtain 5-Arylidene-2-Methylthio-Thioxothiazol-4-one (**39**)

Sodium (0.506 g, 22 mmol) was added to ethanol (34.10 mL). To this solution, the appropriate 5-arylidenethioxothiazolidin-4-one (22 mmol) was added and the reaction mixture was stirred at room temperature for 3 min. Then, iodomethane (3.34 g, 22 mmol) was added and the reaction mixture was stirred for 24 h. The progress of the reaction was monitored by TLC (chloroform: ethyl acetate/1:1). Product was purified by acetone crystallization.

##### (*Z*)-5-((9*H*-Fluoren-2-yl)Methylene)-2-(Methylthio)Thiazol-4(5H)-one (**39**)

(*Z*)-5-((9*H*-Fluoren-2-yl)methylene)-2-thioxothiazolidin-4-one (**31**) (22 mmol, 6.80 g) and iodomethane (22 mmol, 3.34 g) were used. Yellow solid. Yield 99.0 %; mp 202–204 °C. C_18_H_13_NOS_2_ MW 323.43. LC/MS±: (ESI) *m/z* [M + H]^+^ 324.18. ^1^H-NMR (DMSO-d_6_, ppm): δ 8.08–8.00 (d def., 1H, Ar-5-H), 7.99–7.93 (d def., 1H, Ar-4-H), 7.89 (s, 1H, C=CH), 7.86–7.78 (m, 1H, Ar-1-H), 7.71–7.65 (d def., 1H, Ar-3-H), 7.63–7.57 (d def., 1H, Ar-8-H), 7.43–7.31 (m, 2H, Ar-6,7-H), 3.98 (s, 2H, Ar-9-CH_2_), 2.81 (s, 3H, S-CH_3_).

#### 3.1.3. Synthesis of Final Products (**11**, **18**, **22**, **23**) – General Procedure

(*Z*)-5-Arylidene-2-(methylthio)-3*H*-imidazol-4(5*H*)-one or (*Z*)-5-arylidene-2-(methylthio)thiazol-4(5*H*)-one (3–5 mmol) with proper amine (3–6 mmol) was heated and stirred with controlled temperature (120–130 °C) for 15 min. After that time, ethanol (15 mL) was added. Reaction mixture was refluxed for 5–6 h and then stirred at room temperature for 20 h. Then, gaseous hydrochloride acid was used for conversion into hydrochloride forms. If necessary, crystallization using ethanol was performed.

##### (*Z*)-5-(Naphthalen-1-ylmethylene)-2-(4-Morpholinopiperidin-1-yl)-Imidazol-4(*5H*)-one Hydrochloride (**11**)

(*Z*)-5-(Naphthalen-1-ylmethylene)-2-(methylthio)-imidazol-4(*5H*)-one (**38**) (5 mmol, 1.34 g) and 4-morpholinopiperidine (5 mmol, 0.85 g) were used. Yellow solid. Yield 39.26%; mp 244–249 °C. C_23_H_26_N_4_O_2_·3HCl·0.5H_2_O MW 508.87. LC/MS±: purity 96.92% t_R_ = 3.66, (ESI) *m/z* [M + H]^+^ 391.24. ^1^H-NMR (DMSO-d_6_, ppm): δ 11.88 (s, 1H, NH^+^), 8.20 (s, 1H, N3-H), 8.11–8.05 (m, 1H, Ar-8-H), 7.98–7.90 (m, 2H, Ar-4,5-H), 7.62–7.49 (m, 4H, Ar-2,3,6,7-H), 7.25 (s, 1H, CH=C), 4.63–2.90 (m, 13H, Mor, Pp-2,4,6-H), 2.31–2.19 (m, 2H, Pp-3,5-H_b_), 1.89–1.75 (m, 2H, Pp-3,5-H_a_).

##### (*Z*)-5-(Naphthalen-1-ylmethylene)-2-Amino-3-(3-Morpholinopropyl)-Imidazol-4(5*H*)-one Hydrochloride (**18**)

(*Z*)-5-(Naphthalen-1-ylmethylene)-2-(methylthio)-3*H*-imidazol-4(5*H*)-one (**35**) (5 mmol, 1.34 g) and 3-morpholinopropan-1-amine (6 mmol, 0.87 g) were used. Yellow solid. Yield 64.54 %; mp 265–268 °C. C_21_H_25_ClN_4_O_2_ MW 400.90. LC/MS±: purity 99.69% t_R_= 3.56, (ESI) *m/z* [M + H] 365.19. ^1^H-NMR (DMSO-d_6_, ppm): δ 11.54 (br. s, 1H, NH^+^), 9.55 (s, 1H, N3-H), 8.15–8.04 (m, 1H, Ar-8-H), 8.00–7.91 (m, 2H, Ar-4,5-H), 7.84–7.72 (m, 1H, Ar-2-H), 7.65–7.53 (m, 3H, Ar-3,6,7-H) 7.39–7.27 (m, 1H, CH=C), 4.39–2.77 (m, 12H, N3-C*H*_2_-CH_2_-C*H*_2_, Mor), 2.11–1.87 (m, 2H, N3-CH_2_-C*H*_2_). ^13^C-NMR (DMSO-d_6_, ppm): δ 133.79, 133.48, 131.78, 131.61, 129.18, 128.95, 126.06, 126.01, 125.60, 66.59, 53.77, 53.72, 40.79, 40.50, 40.22, 39.94, 39.66, 39.38, 39.09.

##### (*Z*)-5-((9*H*-Fluoren-2-yl)methylene)-2-(4-Morpholinopiperidin-1-yl)Thiazol-4-one Hydrochloride **(22**)

(*Z*)-5-((9*H*-Fluoren-2-yl)methylene)-2-(methylthio)thiazol-4(5*H*)-one (**39**) (3 mmol, 0.97 g) and 4-morpholinopiperidine (3 mmol, 0.51 g) were used. Yellow solid. Yield 2.46 %; mp 244–246 °C. C_26_H_28_ClN_3_O_2_S MW 482.04. LC/MS±: purity 93.63% t_R_= 5.13, (ESI) *m/z* [M + H] 448.33. ^1^H-NMR (DMSO-d_6_, ppm): δ 8.06–7.98 (m, 1H, Ar-5-H), 7.97–7.92 (m, 1H, Ar-4-H), 7.81 (s, 1H, CH=C), 7.70 (s, 1H, Ar-1-H), 7.67–7.63 (d def., 1H, Ar-3-H), 7.61–7.57 (d def., 1H, Ar-8-H), 7.42–7.32 (m, 2H, Ar-6,7-H), 4.03–3.55 (m, 6H, Ar-9-H, Mor-2,6-H), 3.51–2.54 (m, 9H, Mor-3,5-H, Pp-2,4,6-H), 2.16 (br. s, 2H, Pp-3,5-H_b_), 1.67 (br. s, 2H, Pp-3,5-H_a_).

##### (*Z*)-5-((9*H*-Fluoren-2-yl)methylene)-2-((3-Morpholinopropyl)amino)Thiazol-4(5*H*)-one Hydrochloride (**23**)

(*Z*)-5-((9*H*-Fluoren-2-yl)methylene)-2-(methylthio)thiazol-4(5*H*)-one (**39**) (3 mmol, 0.97 g) and 3-morpholinopropan-1-amine (4 mmol, 0.58 g) were used. Yellow solid. Yield 13.44 %; mp 243–245 °C. C_24_H_26_ClN_3_O_2_S MW 456.00. LC/MS±: purity 91.98% t_R_ = 5.05, (ESI) *m/z* [M + H] 420.22. ^1^H-NMR (DMSO-d_6_, ppm): δ 8.03–7.96 (m, 1H, Ar-5-H), 7.94–7.84 (m, 1H, Ar-4-H), 7.83–7.68 (m, 1H, Ar-3-H), 7.67–7.48 (m, 3H, Ar-1,8-H, C2-NH), 7.44–7.18 (m, 3H, Ar-6,7,8-H), 4.01–3.67 (m, 2H, Ar-9-H), 3.67–2.83 (m, 8H, Mor), 2.37–2.07 (m, 4H, N3-C*H*_2_-CH_2_-C*H*_2_), 1.80–1.62 (m, 2H, N3-CH_2_-C*H*_2_). ^13^C-NMR (DMSO-d_6_, ppm): δ 144.31, 144.21, 142.80, 140.70, 133.15, 129.34, 129.15, 128.62, 127.42, 126.33, 125.71, 121.10, 121.06, 66.64, 55.92, 53.75, 40.79, 40.50, 40.22, 39.94, 39.66, 39.38, 39.09, 25.95.

### 3.2. Crystallographic Studies

Single crystals suitable for an X-ray structure analysis were obtained from the mixture of methanol and butan-2-ol by slow evaporation of the solvent at room temperature.

Intensity data was collected using the XtaLAB Synergy-S diffractometer, equipped with the Cu (1.54184 Å) Kα radiation source and graphite monochromator. The phase problem was solved by application of direct methods in SIR-214 [42] and non-hydrogen atoms were refined anisotropically using weighted full-matrix least-squares on F^2^. Refinement and further calculations were carried out in SHELXL-2014 [43]. The hydrogen atoms bonded to carbon atoms were included in the structure at idealized positions and were refined using a riding model with U_iso_(H) fixed at 1.2 U_eq_ of C. Hydrogen atoms attached to nitrogen and oxygen atoms were found from the difference Fourier map and refined without any restraints. For molecular graphics MERCURY [44] program was applied.

**7**: C_20_H_26_ClO_5_N_3_, M_r_ = 423.89, crystal size = 0.05 × 0.08 × 0.56 mm^3^, monoclinic, space group P2_1_/c, a = 16.5221(3) Å, b = 6.9026(1) Å, c = 18.1445(3) Å, V = 2011.85(6) Å^3^, Z = 4, T = 100(2) K, 37915 reflections collected, 3786 unique reflections (R_int_ = 0.0575), R1 = 0.0316, wR2 = 0.0898 [I > 2σ(I)], R1 = 0.0325, wR2 = 0.0914 [all data].

CCDC 2039456 contains the supplementary crystallographic data. These data can be obtained free of charge from The Cambridge Crystallographic Data Centre via www.ccdc.cam.ac.uk/data_request/cif (accessed on 18 February 2021).

### 3.3. Biological Assays

The strains of *S. aureus* and *K. aerogenes* were cultured and then maintained on Columbia agar (bioMérieux, Marcy-l’Étoile, France) or Trypticase Soy Agar II (TSA II; Becton Dickinson, Franklin Lakes, NJ, USA) supplemented with 5% sheep blood, respectively. Cation-adjusted Mueller-Hinton (MH II) broth necessary for the microbiological assays was purchased from bioMérieux (Lyon, France). Antibiotics needed in susceptibility testing: oxacillin, ampicillin, vancomycin, ciprofloxacin, chloramphenicol, doxycycline, norfloxacin, and erythromycin (lactobionate) were obtained from Sigma-Aldrich (St. Louis, MI, USA) or Amdipharm (London, United Kingdom). 5-arylideneimidazolone and 5-arylidenethiaziolone derivatives were dissolved in DMSO (Merck, Stuttgart, Germany) to a concentration of 40 mM and the solutions obtained were stored at -20 °C until used. Furthermore, the following chemical compounds were applied to perform the RTE assay: K_2_HPO_4_ and MgCl_2_ (Sigma-Aldrich, Saint Louis, MO, USA) to prepare potassium phosphate buffer (PPB), CCCP (Sigma-Aldrich, Saint Louis, MO, USA), 1,2’-dNA (TCI-Europe, Zwijndrecht, Belgium), glucose (Sigma-Aldrich, Saint Louis, MO, USA).

#### 3.3.1. Biological Screening in *S. aureus* Clinical Isolates—Susceptibility Testing

Determination of MIC values, i.e., intrinsic antibacterial activity, of products and antibiotics (oxacillin, ampicillin, vancomycin, ciprofloxacin, and erythromycin) in the presence and absence of compounds tested was conducted by the use of a standard 2-fold microdilution method in cation-adjusted Mueller Hinton broth following the Clinical and Laboratory Standards Institute (CLSI) guidelines [45]. The MIC values were detected using an Infinite M200 pro Tecan microplate reader (Tecan^®^ France, SA-Lyon, France). MIC determinations were conducted in 96-well microtiter plates and repeated 3–4 times.

Initially, MICs of a series of imidazolone derivatives were assessed against susceptible (reference) and MDR strains of staphylococci. Next, MIC values of the aforementioned antibiotics were determined in the absence and presence of imidazolone derivatives to assess the chemosensitizing effect of these compounds. The final concentrations of molecules used in the assays were not greater than the values of their respective MICs/4 to ensure that inhibition did not result from intrinsic antibacterial activity of these compounds. 5-arylideneimidazolones and 5-arylidenethiazolones, which precipitated after addition to bacterial suspension in MH II medium, were tested at the highest concentrations at which precipitation was not observed. The final concentration of dimethyl sulfoxide (DMSO) used to prepare solutions of compounds tested never exceeded 2.5% and did not influence bacterial viability. Antibacterial activity of compounds and antibiotics ampicillin, vancomycin, ciprofloxacin, and erythromycin was recorded as the lowest concentration of molecules able to inhibit bacterial growth after 18 h incubation at 37 °C. The incubation of bacterial strains exposed to oxacillin paired with compounds tested was conducted at 35 °C and extended to 24 h due to frequent occurrence of heterogeneity among MRSA strains. The results are presented as activity gain (A) parameter which was calculated as ratio of MIC of a certain antibiotic (MIC_Ant_) to its MIC_Ant+Comp,_ observed in combination with a tested compound (**7**, **12**–**16**, **19**, or **20**; Equation (1)) [25,40].

(1) $$A=\left(\frac{MIC_{Ant}}{MIC_{Ant+Comp}}\right)$$

#### 3.3.2. Molecular Modeling

Compounds were docked to the crystal structure of PBP2a protein (PDB code: 3ZFZ) using Glide [21,46]. Three-dimensional conformations of compounds were generated in LigPrep and docking was performed to the active and allosteric site of this protein: grid centering at S403 and S240, respectively [21,46,47].

To evaluate the mechanism of action of compounds with PBP2a, the compounds which were found to restore oxacillin activity in MRSA 19449 (**10**, **14**–**16**) underwent molecular dynamic simulations. The docking poses receiving the highest scores were used as starting poses for simulations, which were performed in Desmond, using a TIP3P solvent model [48,49]. Each simulation lasted 100 ns.

#### 3.3.3. Biological Screening in *K. aerogenes*—Susceptibility Testing

MIC values of 5-arylideneimidazolone derivatives were determined following CLSI recommendations as in the methods described for *S. aureus* (Section 3.3.1) [45].

#### 3.3.4. Real-Time Efflux Assay

The inhibitory activity of 5-arylideneimidazolone derivatives towards AcrAB-TolC mediated efflux of 1,2’-dNA was detected in *K. aerogenes* strain Ea-289 by using a real-time fluorometric method. In a first step, bacterial cells were loaded with the environment-sensitive fluorescent membrane probe, 1,2’-dNA, in the presence of the EPI carbonyl cyanide *m*-chlorophenylhydrazone (CCCP) which inactivated the pump by dissipating membrane potential. Next, compounds tested were added at the concentration of 100 µM and the efflux was energizing by automated injection of glucose at a dose of 50 µM. The fluorescence intensity was measured in a microplate reader (Tecan) with an excitation wavelength of λ_ex_ = 370 nm and an emission wavelength of λ_em_ = 420 nm. Since the decrease of fluorescence of 1,2’-dNA was observed in the presence of compounds tested, the pre-energization fluorescence intensity was adjusted to 100 relative fluorescence units and the inhibition efficiency (IE) of each compound was calculated according to Equation (2):

(2) $$\mathrm{IE}\;\left[\%\right]=\frac{\Delta i_{1}}{\Delta i_{2}}*100\%$$

where Δ_i1_ reflects the difference between the fluorescence of 1,2’-dNA in the presence and absence of compound tested after the addition of glucose and Δ_i2_ corresponds to the difference between the fluorescence of 1,2’-dNA in the presence and absence of compound tested before the addition of glucose.

### 3.4. ADMET Screening

#### 3.4.1. In Silico Assays

Computer-aided assays to obtain information about the metabolism of compounds were performed using MetaSite 6.0.1 computer program provided by Molecular Discovery Ltd (Hertfordshire, UK) [50]. During calculations MetaSite take into consideration enzyme-substrate recognition (thermodynamic factor) and chemical transformation catalyzed by the enzymes (kinetic factor). The probable sites of metabolism were obtained using various computational models, liver, skin, brain, and cytochromes. Compounds **7**–**23** were analyzed.

#### 3.4.2. In Vitro Assays

The luminescent CYP3A4 P450-Glo™ assay was carried out according to a protocol described previously (Promega, Madison, WI, USA) [51]. The reference compound ketoconazole was obtained from Sigma-Aldrich. Performance of enzymatic reaction was carried out in white polystyrene, flat-bottom Nunc™ MicroWell™ 96-Well Microplate (Thermo Scientific Waltham, MA, USA) and the result was measured with a microplate reader EnSpire in luminescence mode (PerkinElmer, Waltham, MA USA). IC_50_ values were calculated using GraphPad Prism™ software (version 5.01, San Diego, CA, USA). Compounds **15** and **19** were tested at concentrations from 0.01 μM to 25 μM.

## 4. Conclusions

The series of seventeen 5-arylideneimidazolone (**7**–**21**) or 5-arylidenethiazolone (**22**, **23**) derivatives with potential antibiotic adjuvant activity in both, Gram-positive and Gram-negative bacteria, was obtained in 3- or 4-step synthesis. These compounds contain a morpholine moiety at position 2 (**7**–**13**, **22**, **23**) or 3 (**14**–**21**). X-ray crystallographic studies for one member (**7**) provided an insight into structural properties of this group, confirming the expected *Z*-configuration for the 5-arylidene substituent. Two of the compounds, namely **10** and **15**, demonstrated a significant reduction of oxacillin MIC in MRSA 19449 strain. Furthermore, compounds **14**–**16** proved significant activity in various MRSA strains with oxacillin, and even ampicillin in the case of compound **15**. The lack of adjuvant activity in any susceptible strain suggests a molecular mechanism involving an interaction of the compound with PBP2a. Moreover, molecular modeling studies have shown that binding at the allosteric site of PBP2a is the most probable mechanism of the adjuvant action observed in MRSA strains. Although none of the compounds significantly reduced MIC values of antibiotics in *K. aerogenes* strains, several of them exerted a potent 1,2’-dNA efflux inhibition (up to 90 % for compound **19**) in the RTE assay. A recent study has analyzed the molecular and kinetic, e.g., accumulation and efflux, parameters of various fluoroquinolone efflux in *E. coli* expressing different levels of AcrAB [52]. The efflux index (SICAR: Structure Intracellular Concentration Activity Relationship) determined for each fluoroquinolone clearly demonstrates the key role of antibiotic side chains and internal residues of AcrB in the transporter affinity and in the efficiency of drug translocation across the expel channel [52]. Consistently, the adjuvant potential reflects the strength of the affinity AcrB-substrate and depends on the molecular interaction between the partners, e.g., AcrB-antibiotic and AcrB-adjuvant. This suggests potential efflux pump inhibitory (EPI) properties for these 5-arylideneimidazolones.

Summing up, the comprehensive SAR analysis performed points out compound **15** as the most promising due to the ability to enhance both, oxacillin, and ampicillin, in various MRSA strains, and also thanks to its EPI properties. Additionally, this compound shows good drug-like properties in the water solubility and in vitro DDI assays. Based on the results obtained, the *p*-phenylbenzylidene derivative **15** exhibits outstanding MDR-reversal and drug-like properties and is open to further modifications in the search for new and versatile agents to overcome bacterial MDR, especially in Gram-positive and Gram-negative pathogens.

## Figures and Tables

**Table 1:**
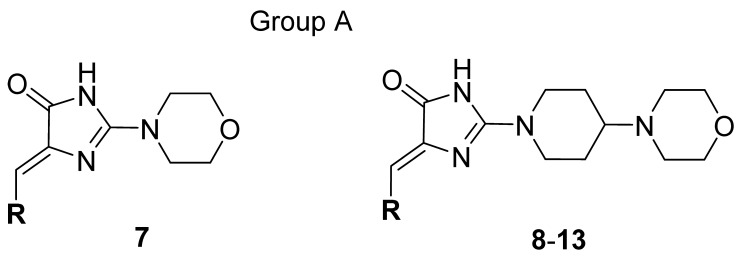
General structure and modifications of group A (**7**–**13**).

Cpd	7, 8	9	10	11	12	13
**R**	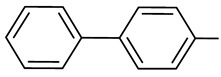	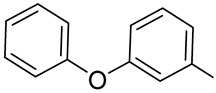	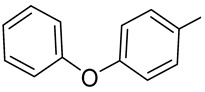	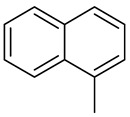	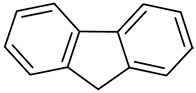	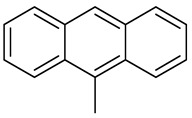

**Table 2:**
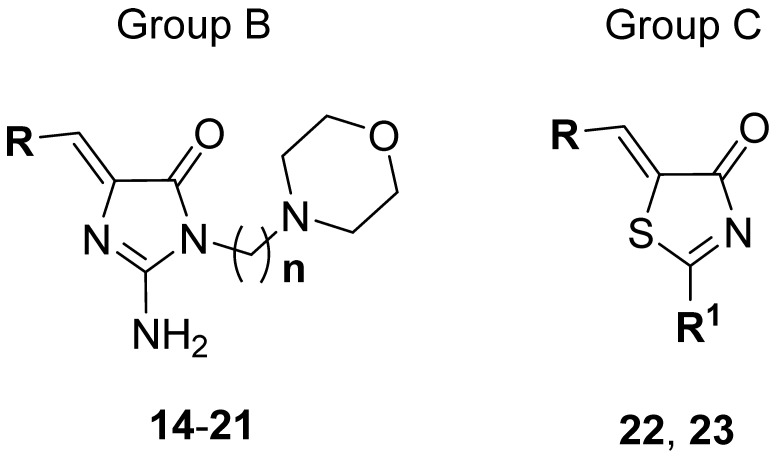
General structures and modifications of group B and C (**14**–**23**).

Cpd	Group	R	n	Cpd	Group	R	n	R^1^
**14**	B	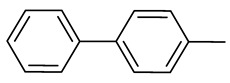	2	**19**	B	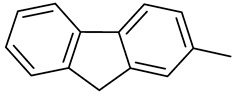	3	-
**15**	B	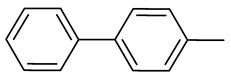	3	**20**	B	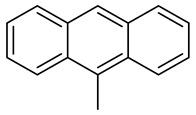	3	-
**16**	B	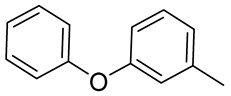	3	**21**	B	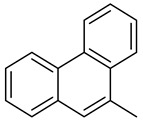	3	-
**17**	B	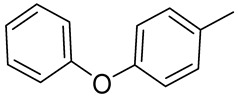	3	**22**	C	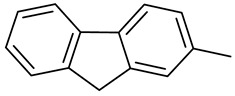	-	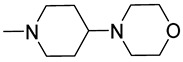
**18**	B	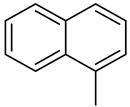	3	**23**	C	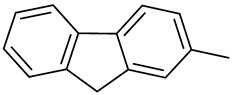	-	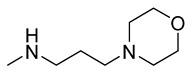

**Table 3:** Parameters of intermolecular hydrogen bonds.

D-H···A	H···A (Å)	D···A (Å)	D-H-A (°)	Symmetry Code
N1-H1N···O1W	2.00(2)	2.875(1)	173(2)	
O1W-H1WA···O2W	2.09(3)	2.921(2)	170(2)	
O2W-H2WB···O3W	1.93(2)	2.712(2)	174(2)	
O3W-H3WA···Cl1	2.44(3)	3.239(1)	167(2)	
N3-H3N···O2W	2.01(2)	2.857(2)	173(2)	x, −y+1/2, z+1/2
O1W-H1WB···Cl1	2.50(3)	3.334(1)	174(2)	−x+1, y+1/2, −z+1/2
O2W-H2WA···Cl1	2.41(2)	3.206(1)	170(2)	−x+1, y−1/2, −z+1/2
O3W-H2WB···Cl1	2.39(3)	3.203(1)	162(2)	−x+1, y+1/2, −z+1/2
C21-H21B···O1W	2.43	3.136(2)	127.5	
C6-H6···O4	2.46	3.151(2)	129.1	−x, −y+1, −z+1
C19-H19A···O2W	2.45	3.433(2)	170.0	x, −y+1/2, z+1/2
C19-H19B···O1	2.32	3.283(2)	163.8	−x+1, −y, −z+1
C20-H20A···Cl1	2.90	3.845(1)	159.5	x, −y+1/2, z+1/2
C20-H20B···O1	2.65	3.295(2)	122.7	−x+1, −y, −z+1
C21-H21A···Cl1	2.96	3.879(1)	155.0	−x+1, y+1/2, −z+1/2

**Table 4:** Ability of compounds (**10** and **15**) to decrease oxacillin MIC in *S. aureus* strains.

Cpd	^1^*S. aureus* ATCC 25923			^1^ MRSA 19449		
	Conc. of cpd [mM]	^2^ MIC Reduction [µg/mL]	^3^ A	Conc. of cpd [mM]	^2^ MIC Reduction [µg/mL]	^3^ A
**10**	0.0625	No effect	1	0.125	From 256 to 64/32	4–8
**15**	0.03125	No effect	1	0.125	From 256 to 128/64	2–4

Abbreviations: Cpd, compound; Conc., concentration; A, activity gain. ^1^ Assays performed in 3–4 repetitions in either, the susceptible (ATCC 25923) or the resistant (MRSA 19449) strains. ^2^ Reduction from MIC of oxacillin tested alone to MIC of oxacillin in combination with **10** and **15**, respectively (top /bottom MIC values noted in 3–4 repetitions). ^3^ Activity gain assessed as the ratio of MIC of oxacillin to MIC of oxacillin in combination with **10** and **15**, respectively (see Equation (1), Section 3.3.1.), the range of A observed due to the MIC differences (top and bottom values) in 3–4 repetitions; A≥4 for active compounds potentiating effects of antibiotics. Detailed results for all compounds tested are presented in Supplementary material (pp. 10–11).

**Table 5:** Antibacterial activity, represented by MIC values, of compounds **7**, **12**–**16**, **19**, **20** against *K. aerogenes* strains (Ea-294, Ea-308, Ea-289, and CM-64).

Compound	Minimal Inhibitory Concentration (MIC) [mM]		
	Ea-294, Ea-308 **	Ea-289 **	CM-64 **
7 *	2	> 2	> 2
12 *	1	> 2	> 2
**13**	2	> 2	> 2
**14**	2	> 2	> 2
15 *	0.125	> 2	> 2
**16**	0.5	> 2	> 2
19 *	0.25	> 2	> 2
20 *	2	> 2	> 2

* Compounds precipitated after addition of MH II. ** *K. aerogenes* strains; Ea-294, Ea-308—devoid of AcrAB; Ea-289, CM-64—with overexpression of AcrAB-TolC with or without change in porin content, respectively.

**Table 6:** Water solubility, presented in mg/mL and mM/L, of compounds **9**–**12**, **15**, **19**.

Compound	Solubility [mg/mL]	Solubility [mM/L]
**9**	1.483	3.168
**10**	2.685	5.735
**11**	20.153	47.288
**12**	0.765	1.645
**15**	87.581	205.502
**19**	8.530	19.467

## Data Availability

CCDC 2039456 contains the supplementary crystallographic data. These data can be obtained free of charge from The Cambridge Crystallographic Data Centre via www.ccdc.cam.ac.uk/data_request/cif (accessed on 18 February 2021).

## Supplementary Materials

The following are available online at https://www.mdpi.com/1422-0067/22/4/2062/s1, Spectral data for compounds (^1^HNMRs and ^13^CNMRs); Table S1. Antibacterial activity of compounds **7**–**23** against *S. aureus* ATCC 25923 (reference) and MRSA 19449 (resistant) strains [1]. Table S2. Effect of 5-arylideneimidazolone and 5-arylidenethiazolone derivatives on the susceptibility of *S. aureus* strains to oxacillin [1]. Table S3. Antibacterial activity of compounds **14**–**16** against various *S. aureus* strains. Table S4. Effect of compounds **14**–**16** on the susceptibility of *S. aureus* MM-O021 and Mu50 strains to oxacillin. Table S5. Effect of compounds **14**–**16** on the susceptibility of *S. aureus* R46-CC22 and R45-CC45 strains to oxacillin. Table S6. Effect of compounds **14–16** on the susceptibility of *S. aureus* MM-O021 and R45-CC45 strains to ampicillin. Table S7. Effect of compounds **14-16** on the susceptibility of *S. aureus* MM-O058, MM-N072, and USA300 LAC strains to ampicillin. Table S8. Effect of compounds **14**–**16** on the susceptibility of *S. aureus* 5328, LG-N017, and R46-CC22 strains to ampicillin. Table S9. Effect of compounds **14**–**16** on the susceptibility of *S. aureus* COL and Mu50 strains to ampicillin. Table S10. Effect of compounds **14**–**16** on the susceptibility of *S. aureus* Mu50 strain to vancomycin. Table S11. Effect of compounds **14**–**16** on the susceptibility of *S. aureus* USA300 LAC, R46-CC22, and Mu50 strains to ciprofloxacin. Table S12. Effect of compounds **14**–**16** on the susceptibility of *S. aureus* MM-O058, MM-N072, and LG-N017 strains to erythromycin. Table S13. Effect of compounds **14**–**16** on the susceptibility of *S. aureus* MM-O021 and USA300 LAC strains to erythromycin. Table S14. Effect of compounds **14**–**16** on the susceptibility of *S. aureus* 53284 and Mu50 strains to erythromycin. Table S15. Antibacterial activity of compounds **7**–**15, 17**–**19, 21**–**23** against *K. aerogenes* strain (EA 289). Table S16. Effect of compounds **7**–**15, 17**–**19, 21**–**23** on the susceptibility of *K.aerogenes* strain (EA 289) to doxycycline and erythromycin. Table S17. Effect of compounds **7, 12**–**16, 19**–**20** on the susceptibility of *K. aerogenes* strains to doxycycline, chloramphenicol, norfloxacin, and erythromycin. Results for compounds **16** and **20** were already published [1]. Table S18. Results of absorbance used for estimation of water solubility of tested compounds (**9**–**12**, **15**, **19**). Table S19. Results of chosen compounds (**9**–**12**, **15**, **19**) stability in acidic conditions. Table S20. Results of chosen compounds (**9**–**12**, **15**, **19**) stability in basic conditions. Figure S1. Docking results to the active site of PBP2a; a) green: **10**, orange: **15**, magenta: **16**, cyan: **14**; b) green: **7**, orange: **8**, magenta: **9**, cyan: **11**; c) green: **12**, orange: **13**, magenta: **17**, cyan: **18**; d) green: **19**, orange: **20**, magenta: **21**, cyan: **22**, yellow: **23**. Figure S2. Docking results of compounds **7**–**23** to the allosteric site of PBP2a; a) green: **10**, orange: **15**, magenta: **16**, cyan: **14**; b) green: **7**, orange: **8**, magenta: **9**, cyan: **11**; c) green: **12**, orange: **13**, magenta: **17**, cyan: **18**; d) green: **19**, orange: **20**, magenta: **21**, cyan: **22**, yellow: **23**. Figure S3. Ligand-protein interaction diagrams obtained during molecular dynamic simulations for compounds **14**–**16** in the active site. Figure S4. Ligand-protein interaction diagrams obtained during molecular dynamic simulations for compounds **14**–**16** in the allosteric site. Figure S5. Results for CYP3A4 metabolism of compound **15** from in silico assay (5 most possible metabolites). Figure S6. Results for CYP3A4 metabolism of compound **19** from in silico assay (5 most possible metabolites).
