# Supplementary material for: Molecular Insights into an Antibiotic Enhancer Action of New Morpholine-Containing 5-Arylideneimidazolones in the Fight against MDR Bacteria
Source: Int J Mol Sci. 2021 Feb 19;22(4):2062. doi: 10.3390/ijms22042062 (PMC7922564; doi:10.3390/ijms22042062)
Supplement: Supplementary file 1 [file ijms-22-02062-s001.pdf]

Supplementary Materials

Molecular insights into an antibiotic enhancer action of new morpholine-containing 5-arylideneimidazolones in the fight against MDR bacteria

Aneta Kaczor [1], Karolina Witek [1-5], Sabina Podlewska [1,6], Veronique Sinou [3], Joanna Czekajewska [2], Ewa Żesławska [7], Agata Doroz-Płonka [1], Annamaria Lubelska [1], Gniewomir Latacz [1], Wojciech Nitek [8], Markus Bischoff [4], Sandrine Alibert [3], Jean-Marie Pagès [3], Claus Jacob [5], Elżbieta Karczewska [2], Jean-Michel Bolla [3] and Jadwiga Handzlik [1,*]

I. Spectral analyses for synthesized compounds…………….………...2

II. Microbiological assays…….……………………………………..…...10

III. Docking studies……………….…………………………….….…17

IV. Molecular dynamic simulations..………………………………………19

V. Water solubility…………………...………………………..………..21

VI. Stability in different pH conditions……………………..………...21

VII. Metabolic stability *in silico*…….………………………………………22

References...………..……..………………………………………………22

I. Spectral analyses for synthesized compounds

¹HNMRs

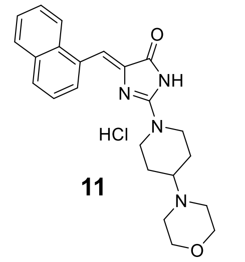

11

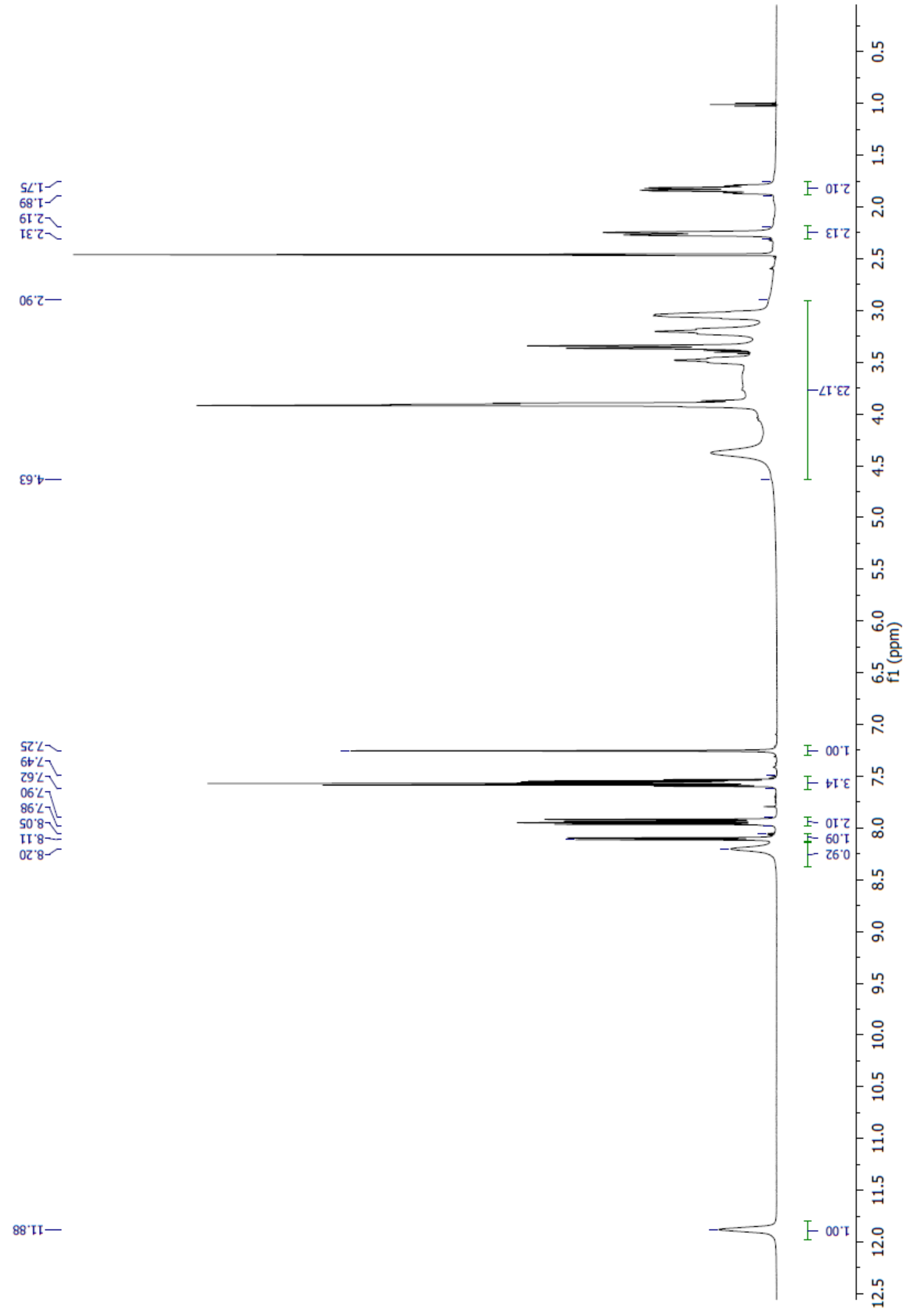

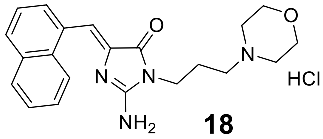

18

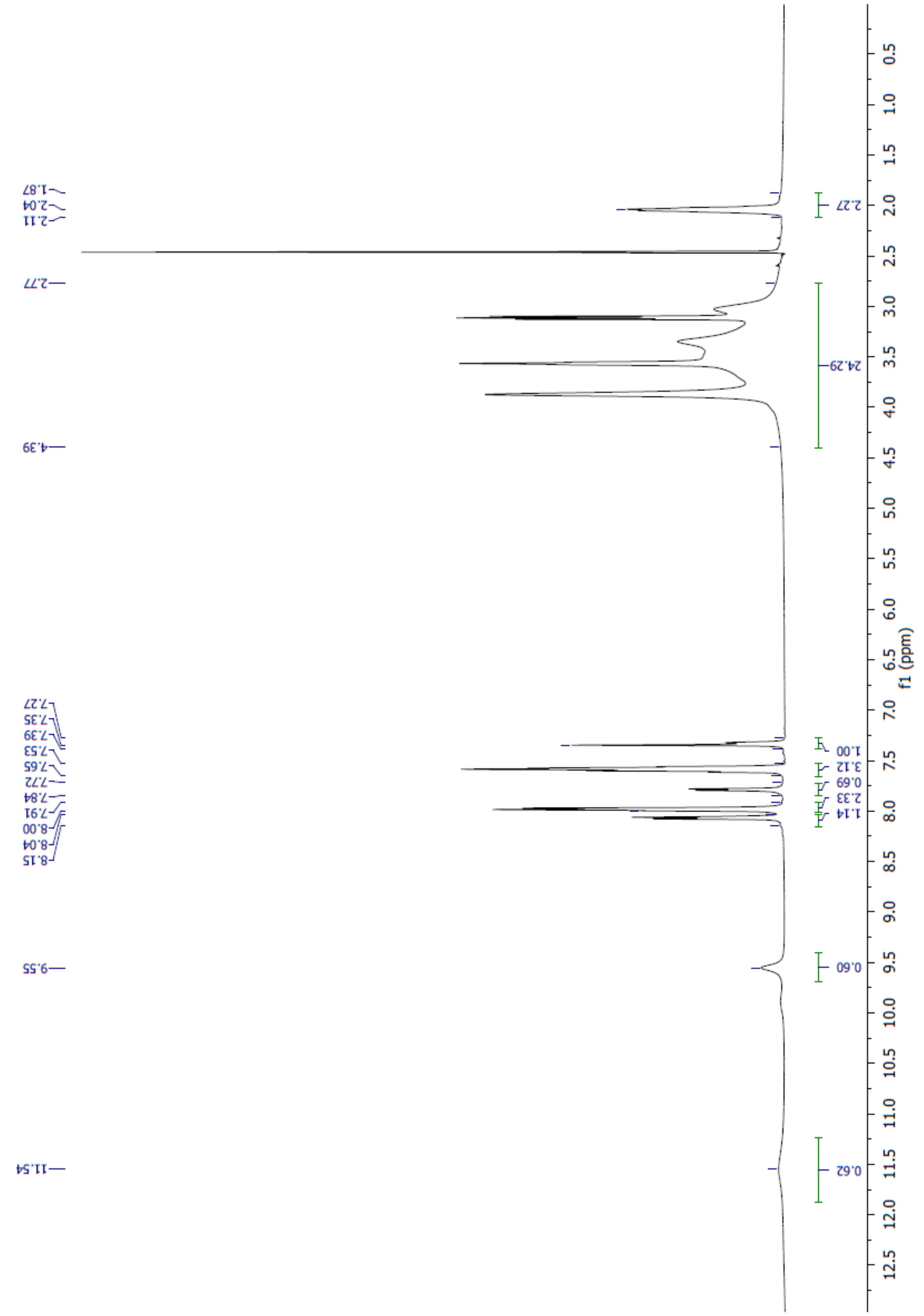

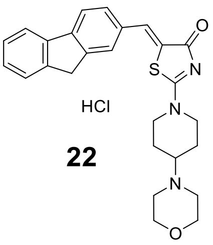

22

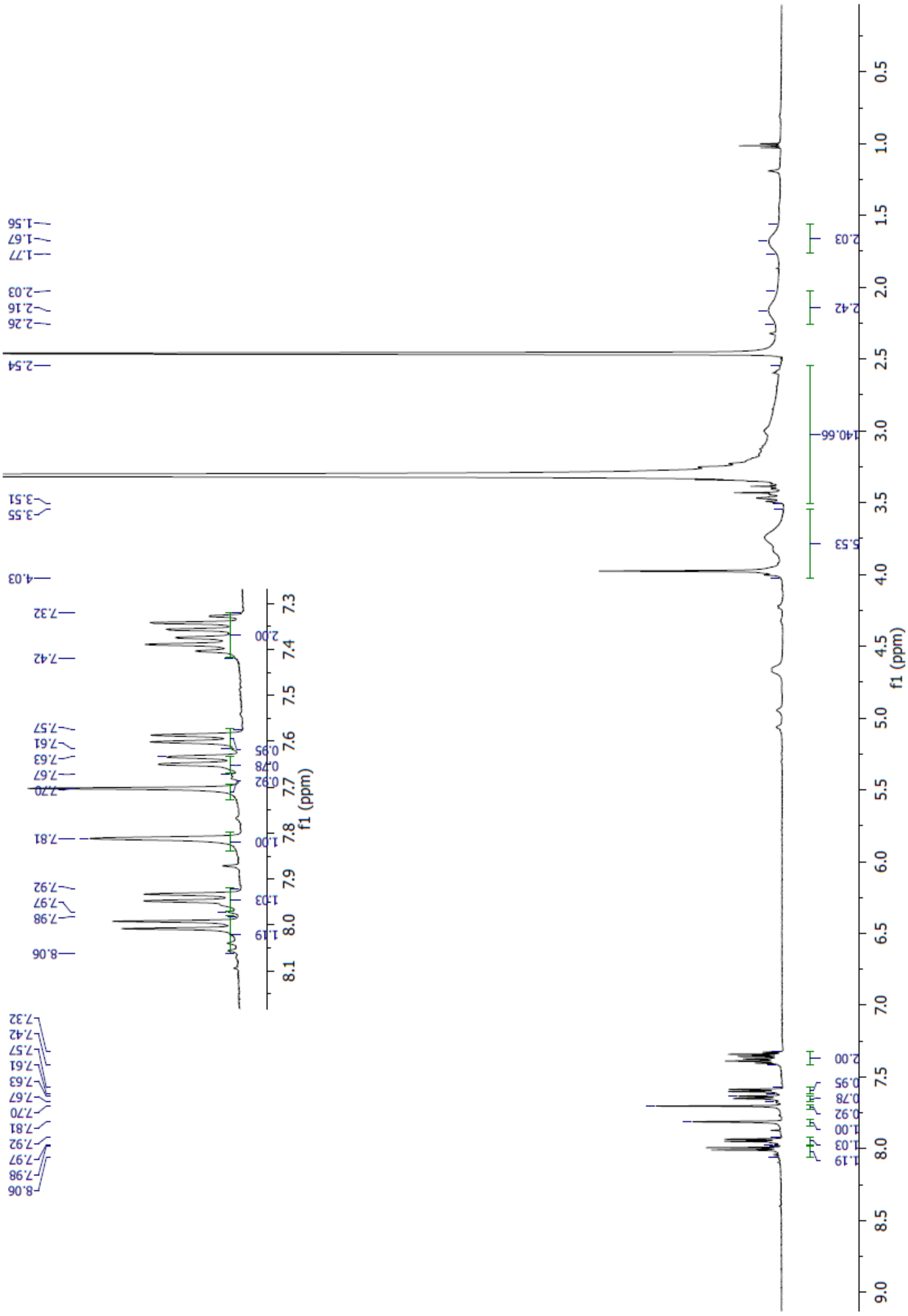

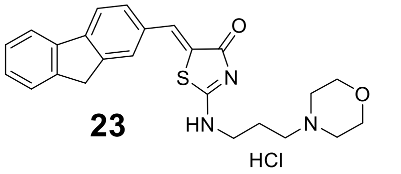
23
HN
S
N
O
N
O
HCl

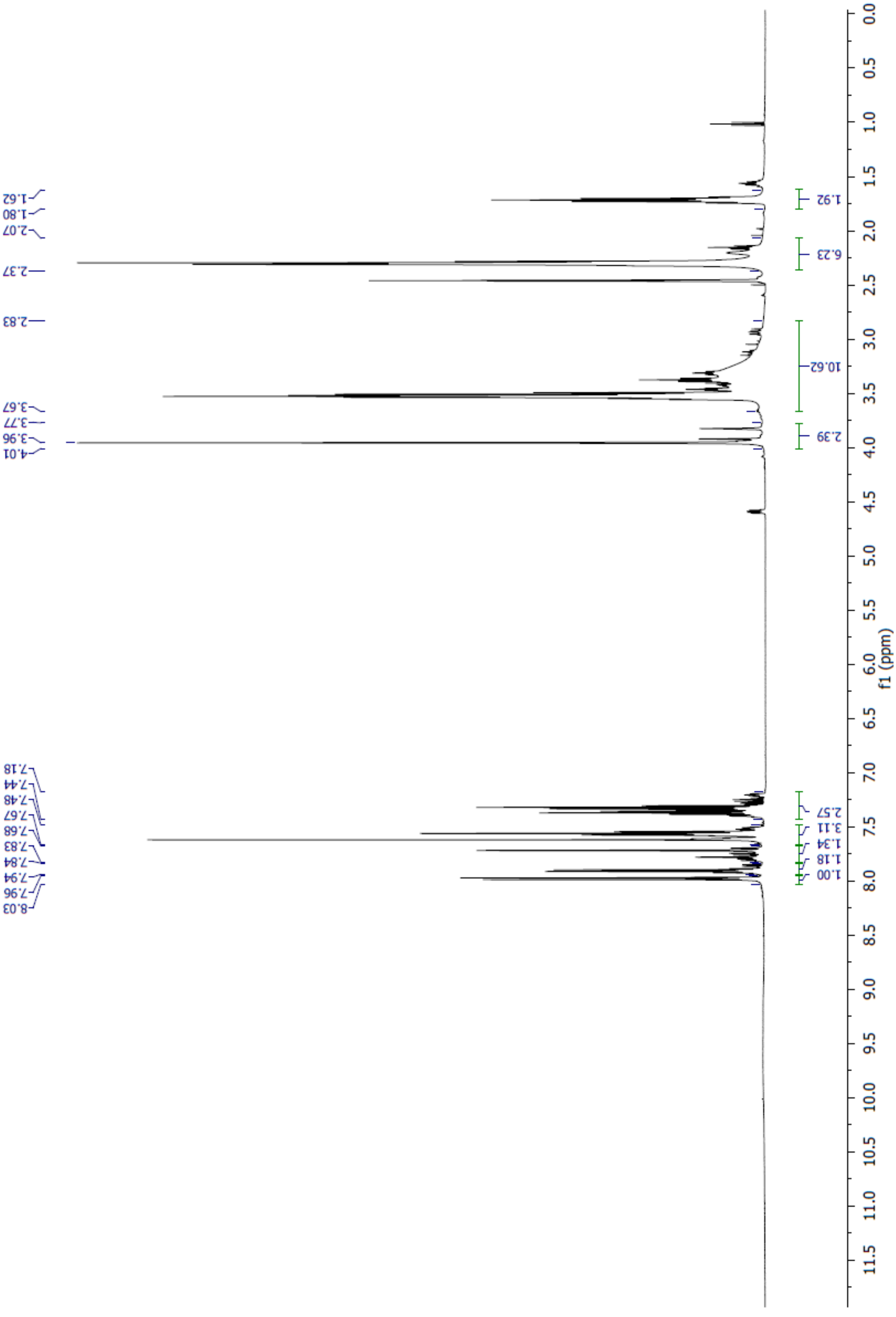
1.62
1.80
2.07
2.37
2.83
3.67
3.77
3.96
4.01
7.18
7.44
7.48
7.67
7.68
7.83
7.84
7.94
7.96
8.03
1.92
6.23
10.62
2.39
2.57
3.11
1.34
1.18
1.00
f1 (ppm)

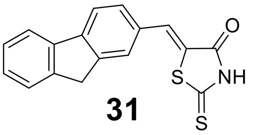

31

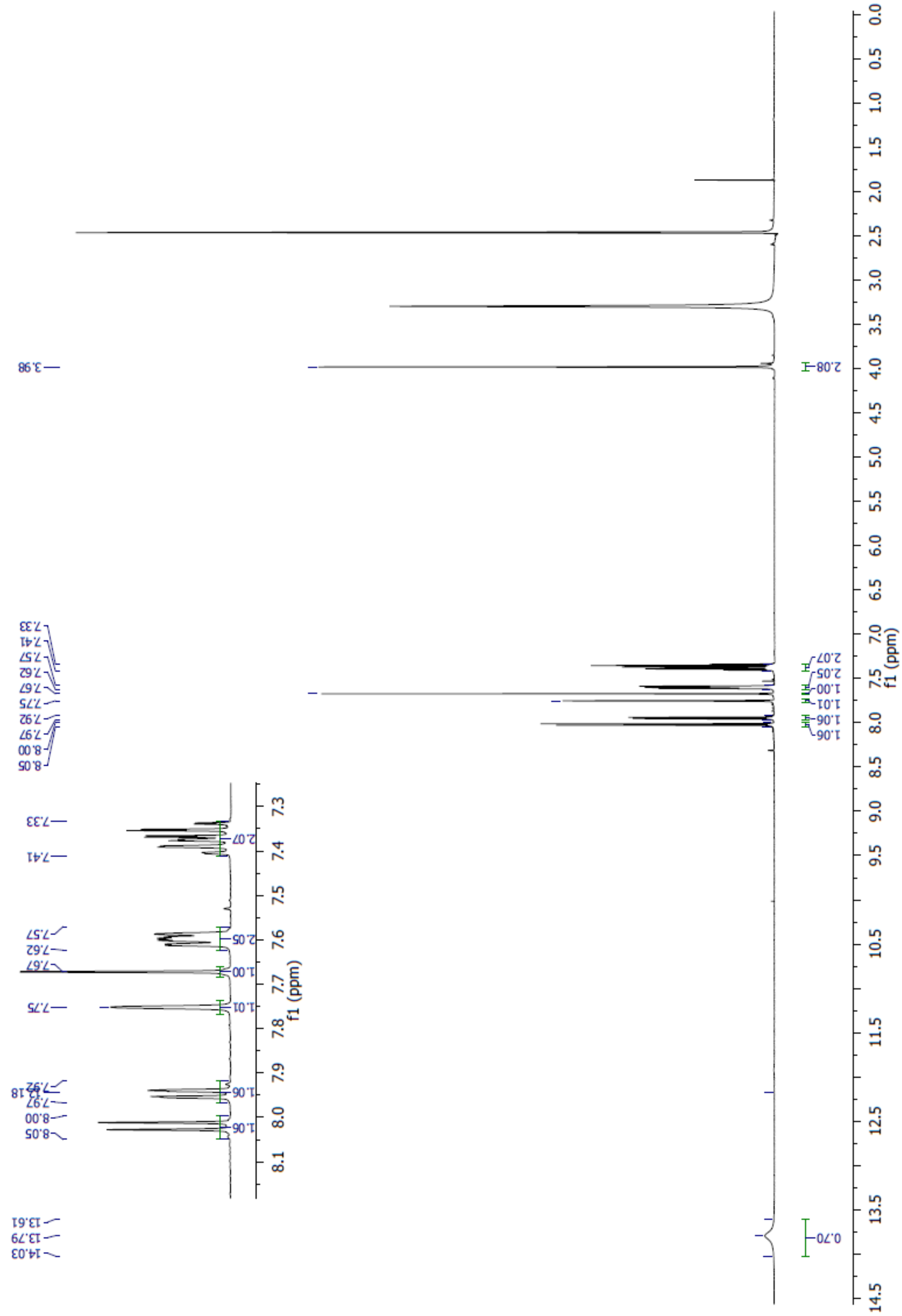

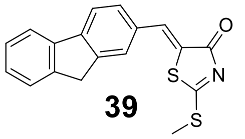

39

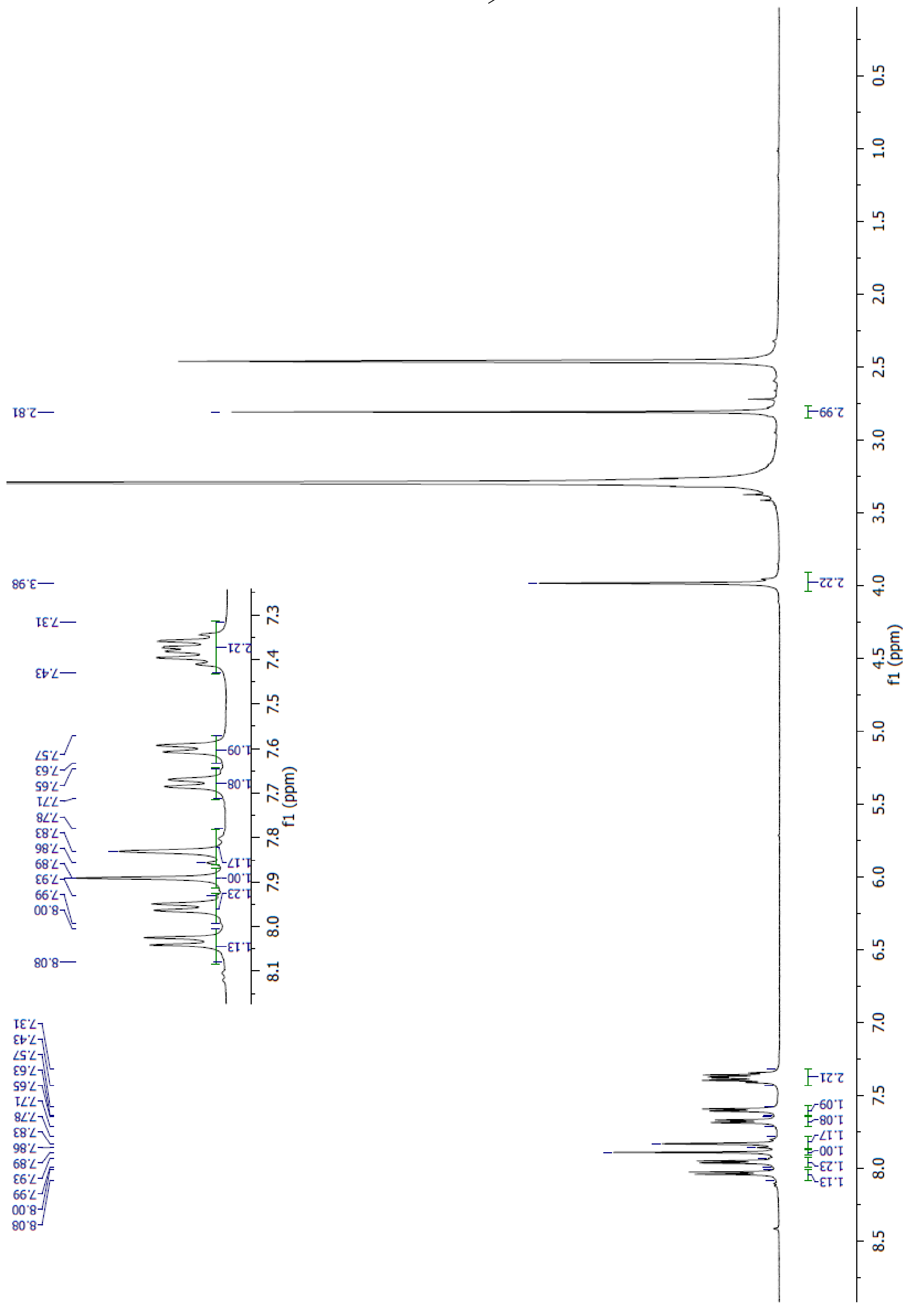

2.81
3.98
7.31
7.43
7.57
7.63
7.65
7.71
7.78
7.83
7.86
7.89
7.93
7.99
8.00
8.08
2.99
2.22
2.21
1.09
1.08
1.17
1.00
1.23
1.13
f1 (ppm)

^{13}CNMRs

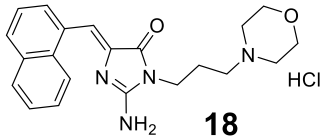
O
N
N
N
O
NH2
HCl

18

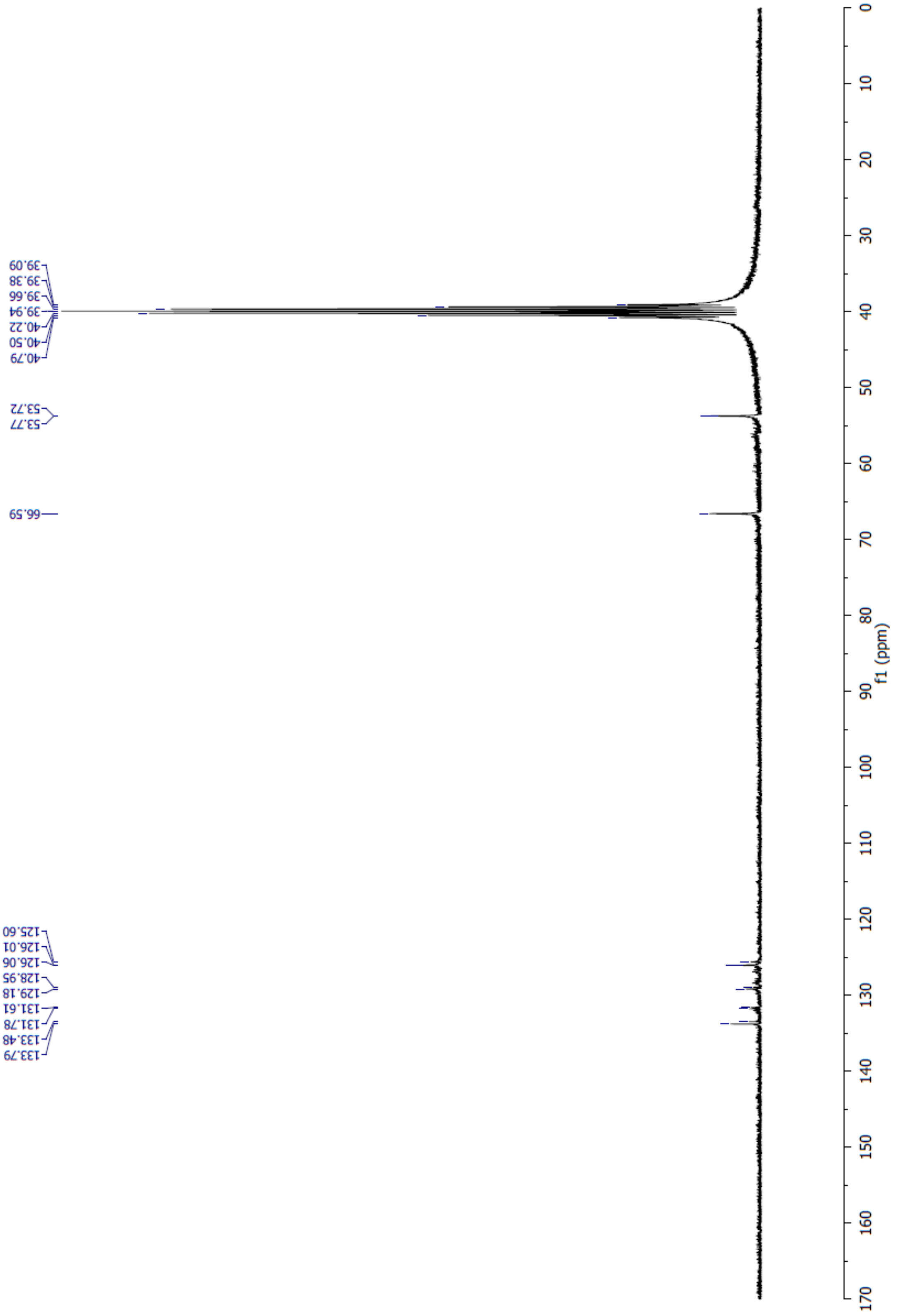
39.09
39.38
39.66
39.94
40.22
40.50
40.79
53.72
53.77
66.59
125.60
126.01
126.06
128.95
129.18
131.61
131.78
133.48
133.79
f1 (ppm)
0
10
20
30
40
50
60
70
80
90
100
110
120
130
140
150
160
170

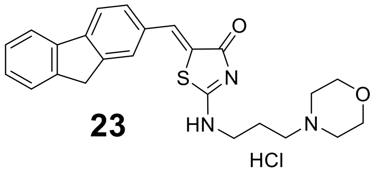
O
S
N
HN
N
O
HCl

23

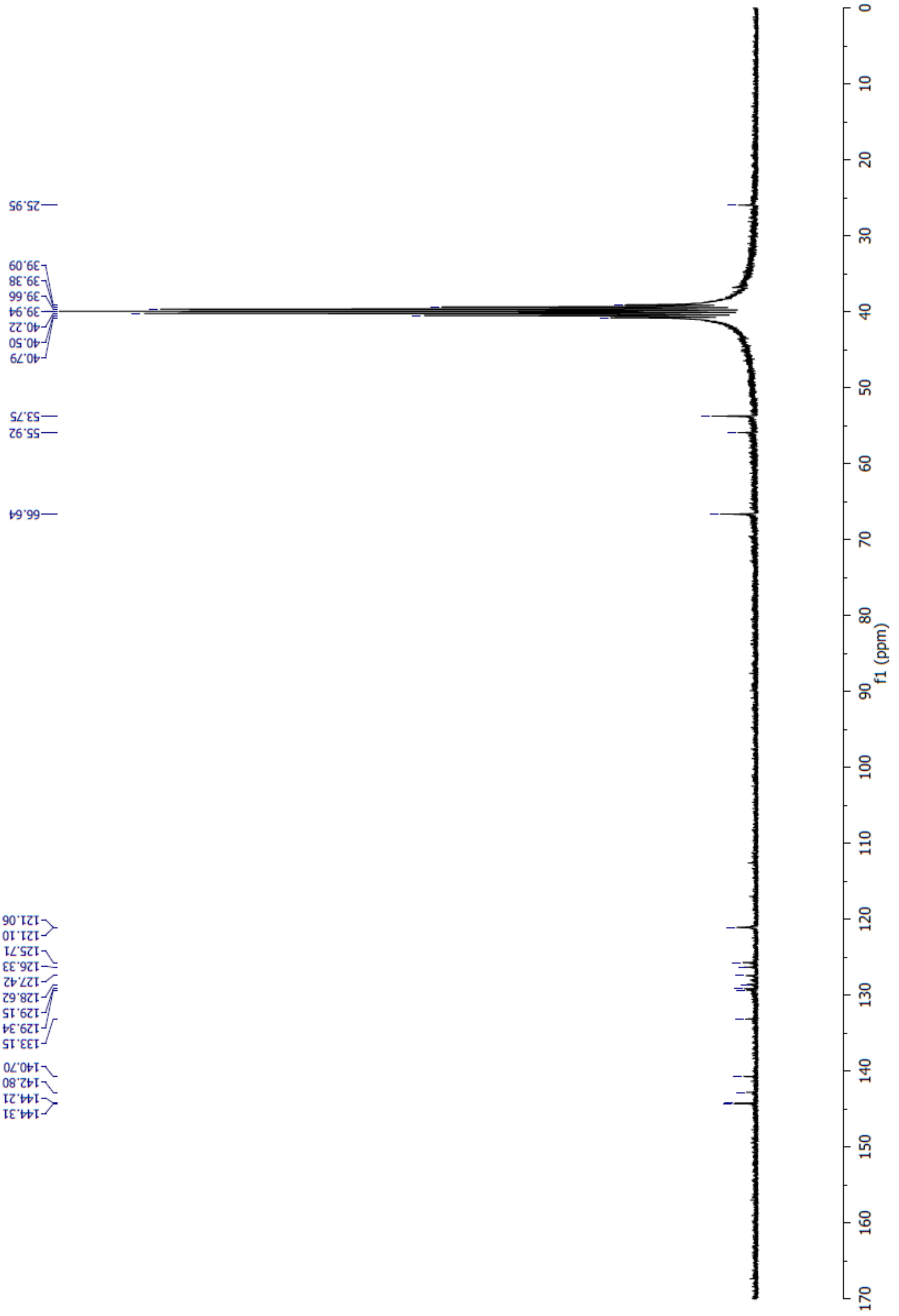
25.95
39.09
39.38
39.66
39.94
40.22
40.50
40.79
53.75
55.92
66.64
121.06
121.10
125.71
126.33
127.42
128.62
129.15
129.34
133.15
140.70
142.80
144.21
144.31
f1 (ppm)

II. Microbiological assays

Table S1. Antibacterial activity of compounds **7-23** against *S. aureus* ATCC 25923 (reference) and MRSA 19449 (resistant) strains [1].

**Compound**	**MIC value/no precipitation [mM]**	
	***S. aureus* ATCC 25923**	**MRSA 19449**
**7**	0.125*	0.125*
**8**	0.25	0.125*
**9**	0.25*	0.25*
**10**	> 0.125	0.125-0.25*
**11**	0.125*	0.125*
**12**	0.03125*	0.03125*
**13**	0.125*	0.125*
**14**	0.0625*	0.0625*
**15**	0.125	0.125-0.25*
**16**	0.125	0.25
**17**	0.125	0.25*
**18**	1	2
**19**	0.0625*	0.0625*
**20**	0.0625*	0.0625*
**21**	0.0625*	0.0625*
**22**	0.0625	0.03125*
**23**	0.03125*	0.03125*

*The highest possible concentration of a compound (due to precipitation) at which growth of bacteria was observed [2].

Table S2. Effect of 5-arylideneimidazolone and 5-arylidenethiazolone derivatives on the susceptibility of *S. aureus* strains to oxacillin [1].

Cpd	[1]*S. aureus* ATCC 25923			[1]MRSA 19449		
	Conc. of cpd [mM]	[2]MICreduction [μg/ml]	A	Conc. of cpd [mM]	[2]MIC reduction [μg/ml]	A
**7**	0.125	No effect	1	0.125	No effect	1
**8**	0.0625	No effect	1	0.125	No effect	1
**9**	0.25	No effect	1	0.25	No effect	1
**10**	0.0625	No effect	1	0.125	From 256 to 64/32	4 - 8
**11**	0.125	No effect	1	0.125	From 256 to 128	2
**12**	0.015625	No effect	1	0.03125	From 256 to 128	2
**13**	0.015625	No effect	1	0.125	No effect	1
**14**	0.0625	No effect	1	0.0625	No effect	1
**15**	0.03125	No effect	1	0.125	From 256 to 128/64	2 - 4
**16**	0.03125	No effect	1	0.0625	No effect	1
**17**	0.03125	No effect	1	0.125	From 256 to 128	1 - 2
**18**	0.25	No effect	1	0.5	From 256 to 128	2
**19**	0.03125	No effect	1	0.015625	No effect	1
**20**	0.0625	No effect	1	0.0625	No effect	1
**21**	0.0625	No effect	1	0.0625	From 256 to 128	2
**22**	0.015625	No effect	1	0.03125	No effect	1
**23**	0.03125	No effect	1	0.03125	No effect	1

Abbreviations: Cpd, compound; Conc., concentration; A, activity gain. [1] Assays performed in 3-4 repetitions in either, the susceptible (ATCC 25923) or the resistant (MRSA 19449) strains. [2] Reduction from MIC of oxacillin tested alone to MIC of oxacillin in combination with a tested compound (top /bottom MIC values noted in 3-4 repetitions). [3] Activity gain assessed as the ratio of MIC of oxacillin to MIC of oxacillin in combination with a tested compound (see Equation 1, Section 3.3.1.), the range of A observed due to the MIC differences (top and bottom values) in 3-4 repetitions; A≥4 for active compounds potentiating effects of antibiotics; A<4 for inactive ones.

Table S3. Antibacterial activity of compounds **14**-**16** against various *S. aureus* strains.

S. aureus strains	MIC value of compounds [mM]		
	Cpd **14**	Cpd **15**	Cpd **16**
MM-O058	≥ 1	0.25	0.25
MM-N072	≥ 0.5	0.25	0.25
USA300 LAC	≥ 0.5	0.125	0.25
5328	≥ 0.5	0.25	0.25
LG-N017	≥ 0.5	0.25	0.25
MM-O021	≥ 0.5	0.25-0.5	0.25
R46-CC22	≥ 0.5	0.25	0.25 - 0.5
R45-CC45	≥ 0.5	0.125 - 0.25	0.25
COL	≥ 0.5	0.25	0.25
Mu50	≥ 0.5	0.25	0.5-0.25

Table S4. Effect of compounds **14-16** on the susceptibility of *S. aureus* MM-O021 and Mu50 strains to oxacillin.

Cpd	[1]*S. aureus* MM-O021			[1]*S. aureus* Mu50		
	Conc. of cpd [mM]	[2]MIC reduction [μg/ml]	[3]A	Conc. of cpd [mM]	[2]MIC reduction [μg/ml]	[3]A
**14**	0.125	No effect	1	0.125	From 256 to 32	8
**15**	0.0625	From 64 to 16	4	0.0625	From 256 to 32	8
**16**	0.0625	From 64 to 16	4	0.125	From 256 to 32	8

Abbreviations: Cpd, compound; Conc., concentration; A, activity gain. [1] Assays performed in 3-4 repetitions. [2] Reduction from MIC of oxacillin tested alone to MIC of oxacillin in combination with a tested compound. [3] Activity gain assessed as the ratio of MIC of oxacillin to MIC of oxacillin in combination with a tested compound (see Equation 1, Section 3.3.1.); A≥4 for active compounds potentiating effects of antibiotics; A<4 for inactive ones.

.

Table S5. Effect of compounds **14-16** on the susceptibility of *S. aureus* R46-CC22 and R45-CC45 strains to oxacillin.

Cpd	[1]*S. aureus* R46-CC22			[1]*S. aureus* R45-CC45		
	Conc. of cpd [mM]	[2]MIC reduction [μg/ml]	[3]A	Conc. of cpd [mM]	[2]MIC reduction [μg/ml]	[3]A
**14**	0.125	No effect	2	0.125	From 4 to 1	4
**15**	0.0625	From 64 to 16	4	0.0625	From 4 to 1	4
**16**	0.125	No effect	2	0.0625	From 4 to 1	4

Abbreviations: Cpd, compound; Conc., concentration; A, activity gain. [1] Assays performed in 3-4 repetitions. [2] Reduction from MIC of oxacillin tested alone to MIC of oxacillin in combination with a tested compound. [3] Activity gain assessed as the ratio of MIC of oxacillin to MIC of oxacillin in combination with a tested compound (see Equation 1, Section 3.3.1.); A≥4 for active compounds potentiating effects of antibiotics; A<4 for inactive ones.

.

Table S6. Effect of compounds **14–16** on the susceptibility of *S. aureus* MM-O021 and R45-CC45 strains to ampicillin.

Cpd	[1]*S. aureus* MM-O021			[1]*S. aureus* R45-CC45		
	Conc. of cpd [mM]	[2]MIC reduction [μg/ml]	[3]A	Conc. of cpd [mM]	[2]MIC reduction [μg/ml]	[3]A
**14**	0.125	No effect	1	0.125	From 48 to 24/12	2-4
**15**	0.125	From 128 to 16/8	8-16	0.0625	From 48 to 12/6	4-8
**16**	0.0625	No effect	1	0.0625	No effect	1

Abbreviations: Cpd, compound; Conc., concentration; A, activity gain. [1] Assays performed in 3-4 repetitions. [2] Reduction from MIC of ampicillin tested alone to MIC of ampicillin in combination with a tested compound (top /bottom MIC values noted in 3-4 repetitions). [3] Activity gain assessed as the ratio of MIC of ampicillin to MIC of ampicillin in combination with a tested compound (see Equation 1, Section 3.3.1.), the range of A observed due to the MIC differences (top and bottom values) in repetitions; A≥4 for active compounds potentiating effects of antibiotics; A<4 for inactive ones.

Table S7. Effect of compounds **14-16** on the susceptibility of *S. aureus* MM-O058, MM-N072, and USA300 LAC strains to ampicillin.

Cpd	[1]*S. aureus* **MM-O058**			[1]*S. aureus* **MM-N072**			[1]*S. aureus* **USA300 LAC**		
	**Conc. of cpd [mM]**	**[2]MIC reduction [μg/ml]**	**[3]A**	**Conc. of cpd [mM]**	**[2]MIC reduction [μg/ml]**	**[3]A**	**Conc. of cpd [mM]**	**[2]MIC reduction [μg/ml]**	**[3]A**
**14**	0.25	No effect	1	0.125	No effect	1	0.125	No effect	1
**15**	0.0625	No effect	1	0.0625	No effect	1	0.0625	From 64 to 32	2
**16**	0.0625	No effect	1	0.0625	No effect	1	0.0625	No effect	1

Abbreviations: Cpd, compound; Conc., concentration; A, activity gain. [1] Assays performed in triplicate. [2] Reduction from MIC of ampicillin tested alone to MIC of ampicillin in combination with a tested compound. [3] Activity gain assessed as the ratio of MIC of ampicillin to MIC of ampicillin in combination with a tested compound (see Equation 1, Section 3.3.1.); A≥4 for active compounds potentiating effects of antibiotics; A<4 for inactive ones.

Table S8. Effect of compounds **14-16** on the susceptibility of *S. aureus* 5328, LG-N017, and R46-CC22 strains to ampicillin.

Cpd	[1]*S. aureus* **5328**			[1]*S. aureus* **LG-N017**			[1]*S. aureus* **R46-CC22**		
	**Conc. of cpd [mM]**	**[2]MIC reduction [μg/ml]**	**[3]A**	**Conc. of cpd [mM]**	**[2]MIC reduction [μg/ml]**	**[3]A**	**Conc. of cpd [mM]**	**[2]MIC reduction [μg/ml]**	**[3]A**
**14**	0.125	From 64 to 32	2	0.125	From 16 to 8	2	0.125	From 32 to 16	2
**15**	0.0625	From 64 to 32	2	0.0625	No effect	1	0.0625	From 32 to 16	2
**16**	0.0625	No effect	1	0.0625	No effect	1	0.125	From 32 to 16	1

Abbreviations: Cpd, compound; Conc., concentration; A, activity gain. [1] Assays performed in triplicate. [2] Reduction from MIC of ampicillin tested alone to MIC of ampicillin in combination with a tested compound. [3] Activity gain assessed as the ratio of MIC of ampicillin to MIC of ampicillin in combination with a tested compound (see Equation 1, Section 3.3.1.); A≥4 for active compounds potentiating effects of antibiotics; A<4 for inactive ones.

Table S9. Effect of compounds **14-16** on the susceptibility of *S. aureus* COL and Mu50 strains to ampicillin.

Cpd	[1]*S. aureus* **COL**			[1]*S. aureus* **Mu50**		
	**Conc. of cpd [mM]**	**[2]MIC reduction [μg/ml]**	**[3]A**	**Conc. of cpd [mM]**	**[2]MIC reduction [μg/ml]**	**[3]A**
**14**	0.125	No effect	1	0.125	No effect	1
**15**	0.0625	No effect	1	0.0625	From 32 to 16	2
**16**	0.0625	No effect	1	0.125	From 32 to 16	2

Abbreviations: Cpd, compound; Conc., concentration; A, activity gain. [1] Assays performed in triplicate. [2] Reduction from MIC of ampicillin tested alone to MIC of ampicillin in combination with a tested compound. [3] Activity gain assessed as the ratio of MIC of ampicillin to MIC of ampicillin in combination with a tested compound (see Equation 1, Section 3.3.1.); A≥4 for active compounds potentiating effects of antibiotics; A<4 for inactive ones.

Table S10. Effect of compounds **14-16** on the susceptibility of *S. aureus* Mu50 strain to vancomycin.

Cpd	[1]*S. aureus* Mu50		
	Conc. of cpd [mM]	[2]MIC reduction [µg/ml]	[3]A
**14**	0.125	No effect	1
**15**	0.0625	No effect	1
**16**	0.0625	No effect	1

Abbreviations: Cpd, compound; Conc., concentration; A, activity gain. [1] Assays performed in triplicate. [2] Reduction from MIC of vancomycin tested alone to MIC of vancomycin in combination with a tested compound. [3] Activity gain assessed as the ratio of MIC of vancomycin to MIC of vancomycin in combination with a tested compound (see Equation 1, Section 3.3.1.); A≥4 for active compounds potentiating effects of antibiotics; A<4 for inactive ones.

Table S11. Effect of compounds **14-16** on the susceptibility of *S. aureus* USA300 LAC, R46-CC22, and Mu50 strains to ciprofloxacin.

Cpd	[1]*S. aureus* USA300 LAC			[1]*S. aureus* R46-CC22			[1]*S. aureus* Mu50		
	Conc. of cpd [mM]	[2]MIC reduction [µg/ml]	[3]A	Conc. of cpd [mM]	[2]MIC reduction [µg/ml]	[3]A	Conc. of cpd [mM]	[2]MIC reduction [µg/ml]	[3]A
**14**	0.125	No effect	1	0.125	No effect	1	0.125	No effect	1
**15**	0.03125	No effect	1	0.0625	No effect	1	0.0625	No effect	1
**16**	0.0625	No effect	1	0.125	No effect	1	0.0625	No effect	1

Abbreviations: Cpd, compound; Conc., concentration; A, activity gain. [1] Assays performed in triplicate. [2] Reduction from MIC of ciprofloxacin tested alone to MIC of ciprofloxacin in combination with a tested compound. [3] Activity gain assessed as the ratio of MIC of ciprofloxacin to MIC of ciprofloxacin in combination with a tested compound (see Equation 1, Section 3.3.1.); A≥4 for active compounds potentiating effects of antibiotics; A<4 for inactive ones.

Table S12. Effect of compounds **14-16** on the susceptibility of *S. aureus* MM-O058, MM-N072, and LG-N017 strains to erythromycin.

Cpd	[1]*S. aureus* MM-O058			[1]*S. aureus* MM-N072			[1]*S. aureus* LG-N017		
	Conc. of cpd [mM]	[2]MIC reduction [µg/ml]	[3]A	Conc. of cpd [mM]	[2]MIC reduction [µg/ml]	[3]A	Conc. of cpd [mM]	[2]MIC reduction [µg/ml]	[3]A
**14**	0.25	No effect	1	0.125	No effect	1	0.125	No effect	1
**15**	0.0625	No effect	1	0.0625	No effect	1	0.0625	No effect	1
**16**	0.0625	No effect	1	0.0625	No effect	1	0.0625	No effect	1

Abbreviations: Cpd, compound; Conc., concentration; A, activity gain. [1] Assays performed in triplicate. [2] Reduction from MIC of erythromycin tested alone to MIC of erythromycin in combination with a tested compound. [3]Activity gain assessed as the ratio of MIC of erythromycin to MIC of erythromycin in combination with a tested compound (see Equation 1, Section 3.3.1.); A≥4 for active compounds potentiating effects of antibiotics; A<4 for inactive ones.

Table S13. Effect of compounds **14-16** on the susceptibility of *S. aureus* MM-O021 and USA300 LAC strains to erythromycin.

Cpd	[1]*S. aureus* MM-O021			[1]*S. aureus* USA300 LAC		
	Conc. of cpd [mM]	[2]MIC reduction [μg/ml]	[3]A	Conc. of cpd [mM]	[2]MIC reduction [μg/ml]	[3]A
**14**	0.125	No effect	1	0.125	No effect	1
**15**	0.125	No effect	1	0.03125	No effect	1
**16**	0.0625	No effect	1	0.0625	No effect	1

Abbreviations: Cpd, compound; Conc., concentration; A, activity gain. [1] Assays performed in triplicate. [2] Reduction from MIC of erythromycin tested alone to MIC of erythromycin in combination with a tested compound. [3] Activity gain assessed as the ratio of MIC of erythromycin to MIC of erythromycin in combination with a tested compound (see Equation 1, Section 3.3.1.); A≥4 for active compounds potentiating effects of antibiotics; A<4 for inactive ones.

Table S14. Effect of compounds **14-16** on the susceptibility of *S. aureus* 53284 and Mu50 strains to erythromycin.

Cpd	[1]*S. aureus* 53284			[1]*S. aureus* Mu50		
	Conc. of cpd [mM]	[2]MIC reduction [μg/ml]	[3]A	Conc. of cpd [mM]	[2]MIC reduction [μg/ml]	[3]A
**14**	0.125	No effect	1	0.125	No effect	1
**15**	0.0625	No effect	1	0.0625	No effect	1
**16**	0.0625	No effect	1	0.0625	No effect	1

Abbreviations: Cpd, compound; Conc., concentration; A, activity gain. [1] Assays performed in triplicate. [2] Reduction from MIC of erythromycin tested alone to MIC of erythromycin in combination with a tested compound. [3] Activity gain assessed as the ratio of MIC of erythromycin to MIC of erythromycin in combination with a tested compound (see Equation 1, Section 3.3.1.); A≥4 for active compounds potentiating effects of antibiotics; A<4 for inactive ones.

Table S15. Antibacterial activity of compounds **7-15, 17-19, 21-23** against *K. aerogenes* strain (EA 289).

Compound	MIC value/no precipitation [mM]	Compound	MIC value/no precipitation [mM]
**7**	> 0.250	**15**	> 0.250
**8**	> 0.250	**17**	> 0.250
**9**	> 0.250	**18**	> 0.250
**10**	> 0.250	**19**	> 0.250
**11**	> 0.250	**21**	> 0.250
**12**	> 0.250	**22**	> 0.250
**13**	> 0.250	**23**	>0.0625
**14**	> 0.250		

Table S16. Effect of compounds **7**-**15, 17**-**19, 21**-**23** on the susceptibility of *K.aerogenes* strain (EA 289) to doxycycline and erythromycin.

Cpd	[1]Doxycycline			[1]Erythromycin		
	Conc. of cpd [mM]	[2]MIC reduction [μg/ml]	[3]A	Conc. of cpd [mM]	[2]MIC reduction [μg/ml]	[3]A
**7**	0.100	No effect	1	0.100	No effect	1
**8**	0.100	No effect	1	0.100	No effect	1
**9**	0.100	No effect	1	0.100	No effect	1
**10**	0.100	No effect	1	0.100	No effect	1
**11**	0.100	No effect	1	0.100	No effect	1
**12**	0.100	No effect	1	0.100	No effect	1
**13**	0.100	No effect	1	0.100	No effect	1
**14**	0.100	No effect	1	0.100	No effect	1
**15**	0.100	No effect	1	0.100	No effect	1
**17**	0.100	No effect	1	0.100	No effect	1
**18**	0.100	No effect	1	0.100	No effect	1
**19**	0.100	No effect	1	0.100	No effect	1
**21**	0.100	No effect	1	0.100	No effect	1
**22**	0.100	No effect	1	0.100	No effect	1
**23**	0.050	No effect	1	0.050	No effect	1

Abbreviations: Cpd, compound; Conc., concentration; A, activity gain. [1] Assays performed in triplicate. [2] Reduction from MIC of certain antibiotic to its MIC in combination with a tested compound. [3] Activity gain assessed as the ratio of MIC of certain antibiotic to its MIC in combination with a tested compound (see Equation 1, Section 3.3.1.); A≥4 for active compounds potentiating effects of antibiotics; A<4 for inactive ones.

Table S17. Effect of compounds **7, 12**-**16, 19**-**20** on the susceptibility of *K. aerogenes* strains to doxycycline, chloramphenicol, norfloxacin, and erythromycin. Results for compounds **16** and **20** were already published [1].

Cpd	[1]Ea-294, Ea-308			[1]Ea-289			[1]CM-64		
	Conc. of cpd [mM]	[2]MIC reduction [μg/ml]	[3]A	Conc. of cpd [mM]	[2]MIC reduction [μg/ml]	[3]A	Conc. of cpd [mM]	[2]MIC reduction [μg/ml]	[3]A
**7**	0.25	No effect	1	0.25	No effect	1	0.25	No effect	1
**12**	0.125	No effect	1	0.125	No effect	1	0.125	No effect	1
**13**	0.125	No effect	1	0.125	No effect	1	0.125	No effect	1
**14**	0.125	No effect	1	0.125	No effect	1	0.125	No effect	1
**15**	0.03125	No effect	1	0.125	No effect	1	0.125	No effect	1
**16**	0.25	No effect	1	0.25	No effect	1	0.25	No effect	1
**19**	0.0625	No effect	1	0.125	No effect	1	0.125	No effect	1
**20**	0.125	No effect	1	0.125	No effect	1	0.125	No effect	1

Abbreviations: Cpd, compound; Conc., concentration; A, activity gain. [1] Assays performed in triplicate. [2] Reduction from MIC of certain antibiotic to its MIC in combination with a tested compound. [3] Activity gain assessed as the ratio of MIC of antibiotic to MIC of antibiotics in combination with a tested compound (see Equation 1, Section 3.3.1.); A≥4 for active compounds potentiating effects of antibiotics; A<4 for inactive ones.

III. Docking studies

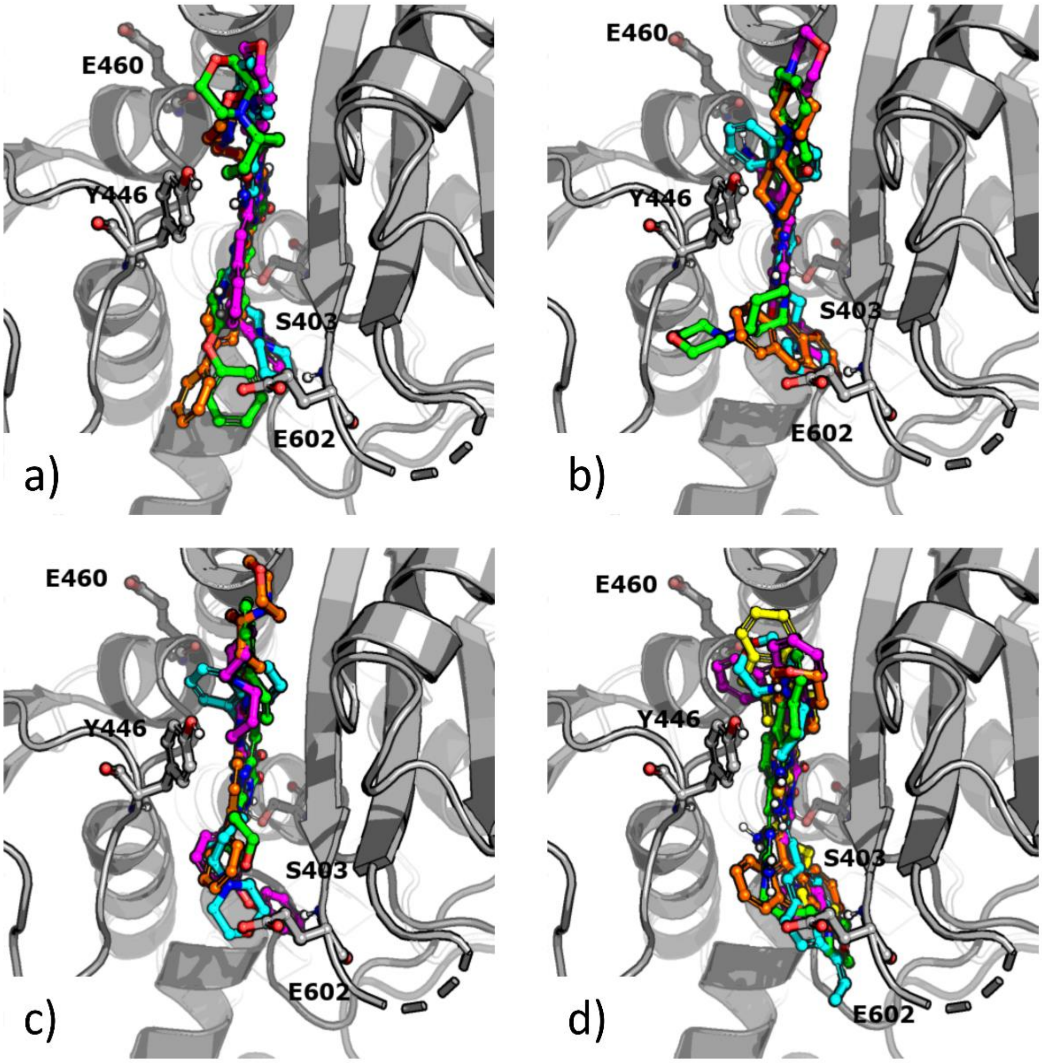

Figure S1. Docking results to the active site of PBP2a; a) green: **10,** orange: **15,** magenta: **16**, cyan: **14**; b) green: **7**, orange: **8**, magenta: **9**, cyan: **11**; c) green: **12,** orange: **13**, magenta: **17**, cyan: **18**; d) green: **19**, orange: **20**, magenta: **21**, cyan: **22**, yellow: **23**.

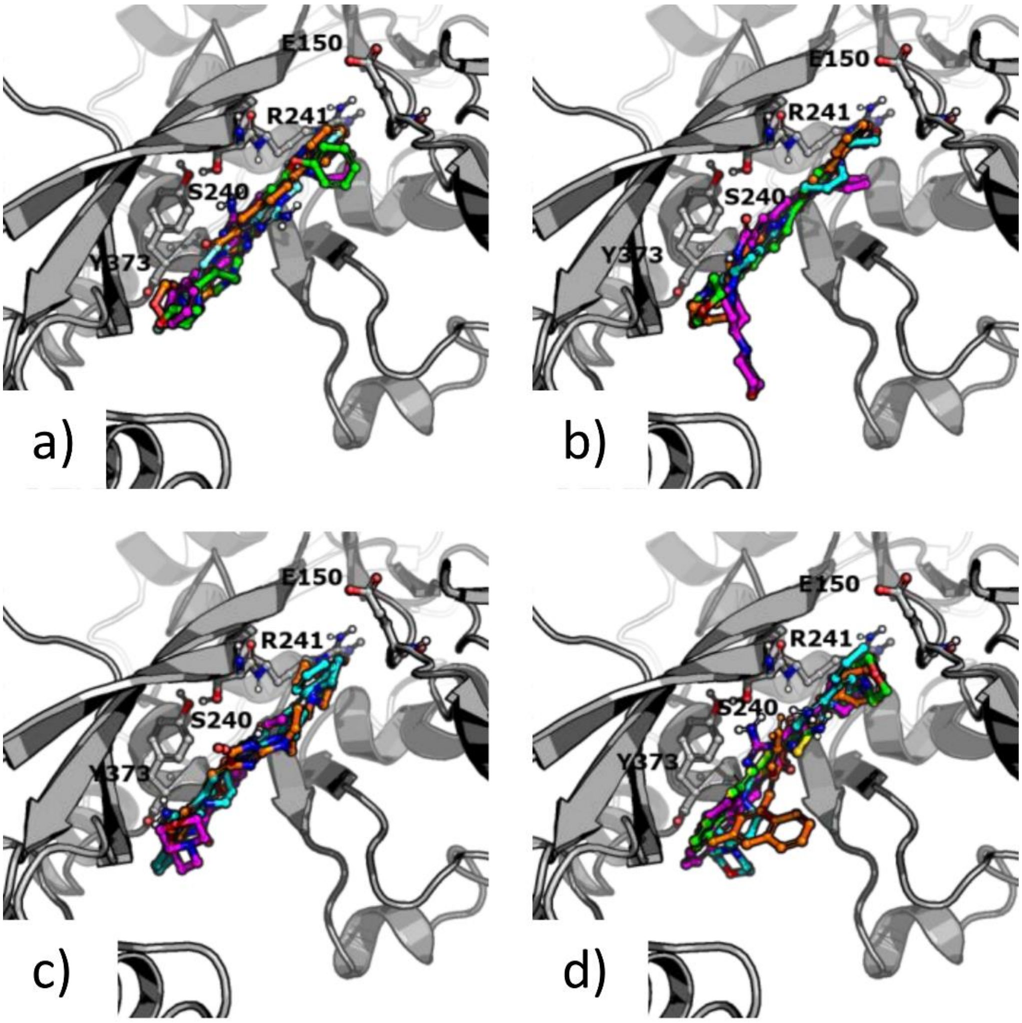

Figure S2. Docking results of compounds **7**-**23** to the allosteric site of PBP2a; a) green: **10**, orange: **15,** magenta: **16**, cyan: **14**; b) green: **7**, orange: **8**, magenta: **9**, cyan: **11**; c) green: **12**, orange: **13**, magenta: **17**, cyan: **18**; d) green: **19**, orange: **20**, magenta: **21**, cyan: **22**, yellow: **23**.

IV. Molecular dynamic simulations

14

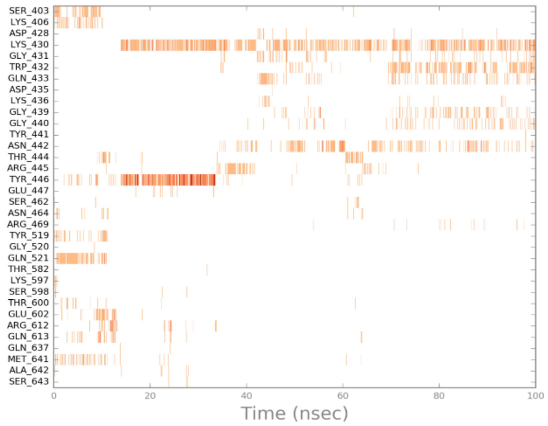

15

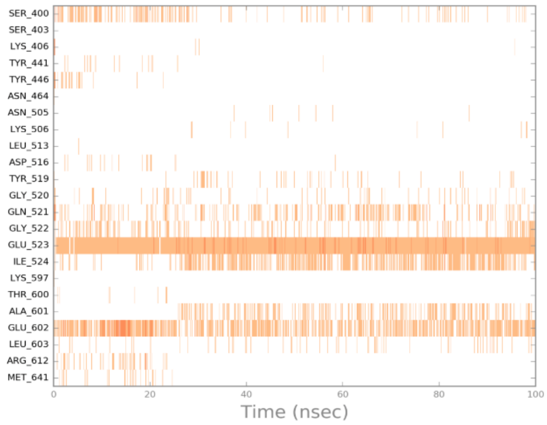

16

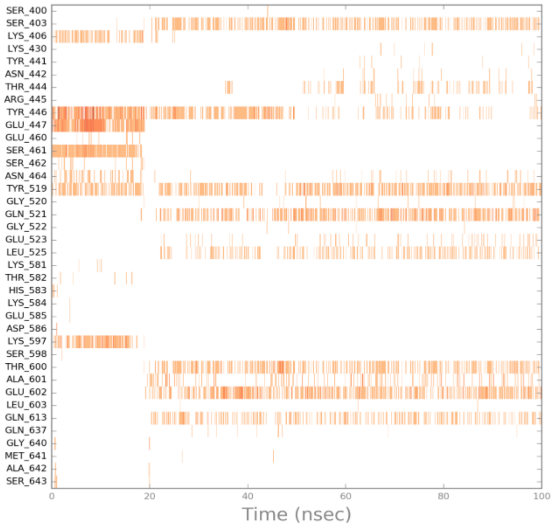

Figure S3. Ligand-protein interaction diagrams obtained during molecular dynamic simulations for compounds **14-16** in the active site.

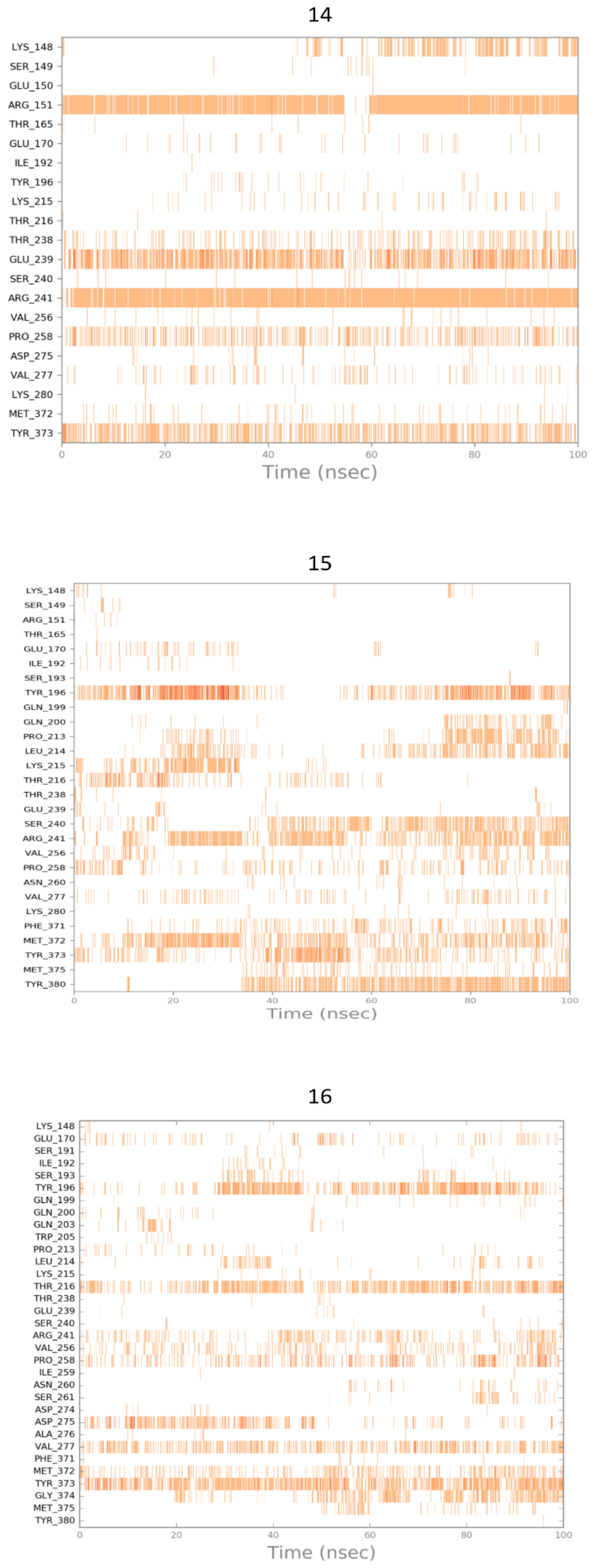

Figure S4. Ligand-protein interaction diagrams obtained during molecular dynamic simulations for compounds **14**-**16** in the allosteric site.

V. Water solubility

Water solubility was obtained using UV spectroscopy method. Absorbance of tested solutions of compounds **9-12**, **15**, and **19,** respectively in suitable dilution, in three repetitions as well as calculated arithmetic means, solubility and standard deviations are presented in Table S18. the solubility results in both, mg/mL and mmol/L, are presented in Table 6 (Section 2.5.1.).

Table S18. Results of absorbance used for estimation of water solubility of tested compounds (**9-12**, **15**, **19**).

Compound	9	10	11	12	15	19
**A_1**	1,52383	1,00518	1,00197	0,81529	1,43112	1,52245
**A_2**	1,52498	1,00571	1,00551	0,81396	1,42902	1,52217
**A_3**	1,52408	1,00794	1,00681	0,81418	1,43256	1,52815
**A_{mean}**	1,524297	1,006277	1,004763	0,814477	1,4309	1,524257
**s**	0,000605	0,001465	0,002505	0,000713	0,00178	0,003375
**S [mg/nL]**	1.483	2.685	20.153	0.765	87.581	8.530
**±SD**	0.00	0.00	0.05	0.00	0.11	0.02

Abbreviations: A_1, A_2,and A_3, UV absorbance in first, second, and third repetition; A_{mean}, arithmetic mean of absorbance from three repetitions; s, standard deviation for absorbance; S, water solubility; SD – standard deviation for solubility.

VI. Stability in different pH conditions

Stability of chosen compounds (**9-12**, **15**, and **19**) were determined using the TLC method. Assays were performed in acidic and basic conditions and were carried out for 48 h. Samples were taken at six-time intervals (10 min., 1 h, 2 h, 3 h, 24 h, 48 h). Results are presented in Tables S18 and S19 as number of spots observed on TLC, where 1 indicate that compound is stable (only TLC spot for proper compound was observed). Higher values point out how many products of degradation was observed, which can be calculating by subtracting one from number of spots.

Table S19. Results of chosen compounds (**9-12**, **15**, **19**) stability in acidic conditions.

Compound	Number of TLC spots in defined time					
	10 min	1 h	2 h	3 h	24 h	48 h
**9**	2	2	2	2	2	2
**10**	1	1	1	1	1	2
**11**	1	1	1	1	1	2
**12**	1	1	1	1	1	1
**15**	2	2	2	2	2	2
**19**	1	1	1	2	2	3

Table S20. Results of chosen compounds (**9-12**, **15**, **19**) stability in basic conditions.

Compound	Number of TLC spots in defined time					
	10 min	1 h	2 h	3 h	24 h	48 h
**9**	2	2	2	2	2	2
**10**	1	1	1	1	1	2
**11**	1	2	2	2	2	2
**12**	7	6	5	4	3	3
**15**	1	1	1	1	1	2
**19**	4	4	4	4	4	4

VII. Metabolic stability *in silico*

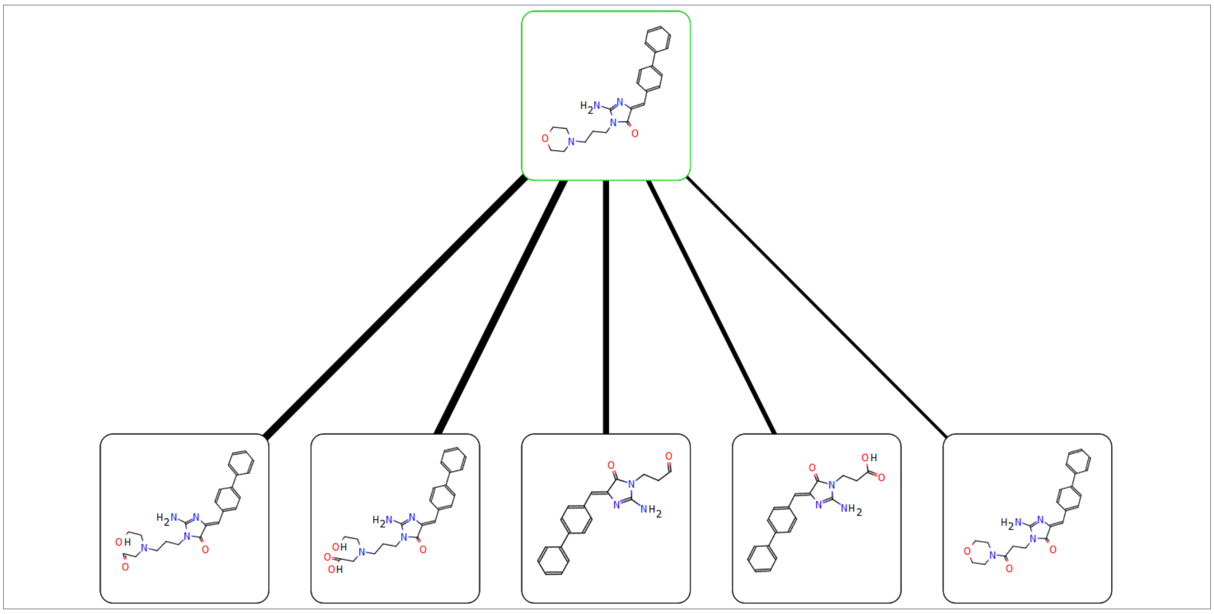

Figure S5. Results for CYP3A4 metabolism of compound **15** from *in silico* assay (5 most possible metabolites).

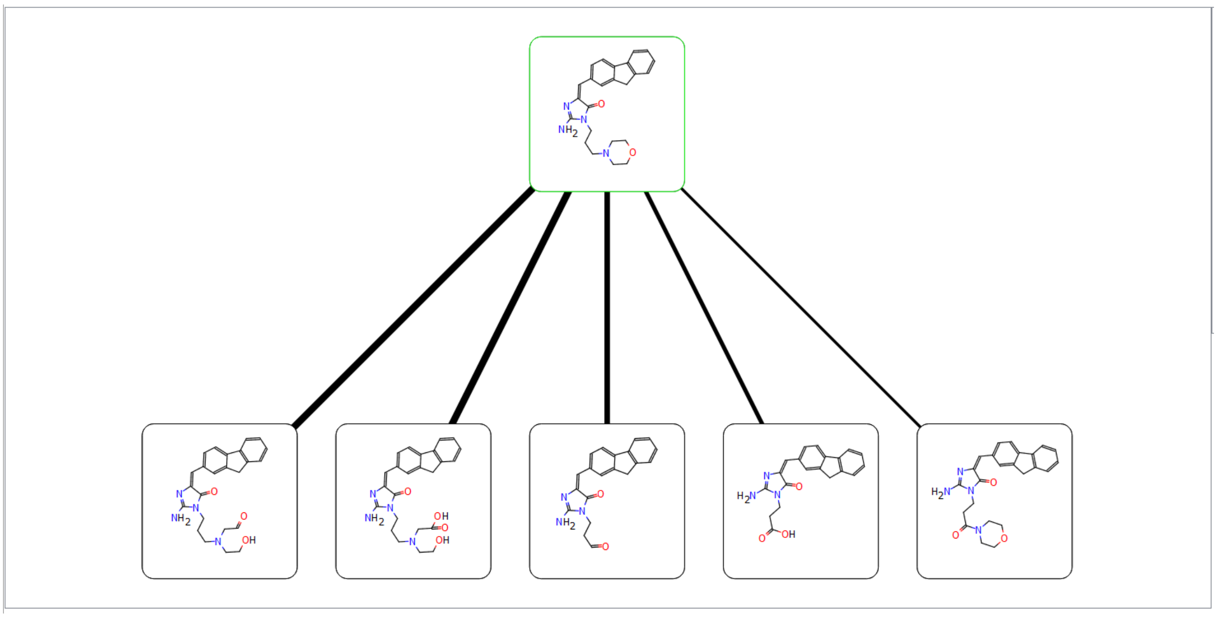

Figure S6. Results for CYP3A4 metabolism of compound **19** from *in silico* assay (5 most possible metabolites).

References:

1. Kaczor, A.; Witek, K.; Podlewska, S.; Czekajewska, J.; Lubelska, A.; Żesławska, E.; Nitek, W.; Latacz, G.; Alibert, S.; Pagès, J.M.; Karczewska, E.; Kieć-Kononowicz, K.; Handzlik, J. 5-Arylideneimidazolones with Amine at Position 3 as Potential Antibiotic Adjuvants against Multidrug Resistant Bacteria. *Molecules.* **2019**, *24*, 438. DOI: 10.3390/molecules24030438.
2. Witek, K.; Latacz, G.; Kaczor, A.; Czekajewska, J.; Żesławska, E.; Chudzik, A.; Karczewska, E.; Nitek, W.; Kieć-Kononowicz, K.; Handzlik, J. Phenylpiperazine 5,5-Dimethylhydantoin Derivatives as First Synthetic Inhibitors of Msr(A) Efflux Pump in Staphylococcus Epidermidis. *Molecules* **2020**, *25*, doi:10.3390/molecules25173788.
